# Tumor-intrinsic ENO1 inhibition promotes antitumor immune response and facilitates the efficacy of anti-PD-L1 immunotherapy in bladder cancer

**DOI:** 10.1186/s13046-025-03464-x

**Published:** 2025-07-15

**Authors:** Chengquan Shen, Jing Liu, Ding Hu, Changxue Liu, Fei Xie, Yonghua Wang

**Affiliations:** 1 Department of Urology, The Affiliated Hospital of Qingdao University, No.16, Jiangsu Road, Qingdao, Shandong 266000 People’s Republic of China; 2 Department of Research Management and International Cooperation, The Affiliated Hospital of Qingdao University, Qingdao, Shandong China; 3 Qingdao Clinical Medical Research Center for Urinary System Diseases, Qingdao, Shandong China; 4 Shandong Province Medical and Health Key Laboratory of Urology, Qingdao, Shandong China

**Keywords:** ENO1, Bladder cancer, SPP1, CD8^+^ T cell, Tumor associated macrophage

## Abstract

Immunotherapy has revolutionized cancer treatment, yet understanding immunotherapy resistance mechanisms remains challenging. Here, a CRISPR cas9 screening in vivo and an RNA-sequencing for clinical immunotherapy resistance BC samples identified enolase 1 (ENO1) as a potent regulator of anti-PD-L1 treatment efficacy. Investigation of clinical BC samples demonstrated a correlation between ENO1 overexpression and immune evasion in BC, evidenced by reduced CD8^+^ T cell infiltration and resistance to anti-PD-L1 therapy. Increased CD8^+^ T cell infiltration and function were indicative of antitumor immunity, which was elicited by ENO1 knockdown, which also suppressed carcinogenesis. Single-cell RNA sequencing demonstrated that wild-type (WT) and ENO1 knockout (KO) tumors have different immune cell compositions with the latter preferring an immunostimulatory microenvironment. Mechanistically, ENO1 regulated CD8^+^ T cell function and tumor-associated macrophage (TAM) polarization via the SPP1-ITGA4/ITGB1 pathway in the TME. Importantly, genetic and pharmacological inhibition of ENO1 sensitizes tumors to anti-tumor immunity and synergizes with anti-PD-L1 therapy. The results highlight tumor-intrinsic ENO1 as a critical regulator of tumor immune evasion in BC. Targeting ENO1 enhance the efficacy of immune checkpoint blockade therapy by promoting antitumor immunity.

**Supplementary Information:**

The online version contains supplementary material available at 10.1186/s13046-025-03464-x.

## Introduction

Bladder cancer (BC) is a prevalent urological malignancy globally, ranking in the top 9. In 2022, there were 613,791 newly diagnosed cases of BC, resulting in 220,349 deaths [1]. Despite the implementation of various innovative treatment approaches for BC, the majority of patients ultimately succumbs to treatment failure caused by drug resistance. Over the past decade, the development of immunotherapy has encouraged improvements in survival rates and fundamentally transformed the approach to treating patients with BC [2]. Nevertheless, resistance to immune checkpoint inhibitors (ICIs) is evident in a significant proportion of patients, underscoring the urgent need to explore potential mechanisms and identify novel treatment targets.

The effectiveness of cancer immunotherapy is greatly influenced by the tumor microenvironment (TME), where a complex interaction occurs among tumor cells, immune cells, and the surrounding factors [3, 4]. This interaction, established through crosstalk between tumor and immune cells, shapes the dynamic and intricate TME. Key components in this microenvironment include immune-suppressive cells like regulatory T cells, myeloid-derived suppressor cells, and tumor-associated macrophages (TAMs), which can hinder effector T cells and natural killer cell activity, thereby dampening the immune response against the tumor [5, 6]. Additionally, increased expression of immune checkpoint molecules such as PD-L1 within the TME by tumor cells can impair T cell function, often leading to immunotherapy resistance [7]. Hence, it is crucial to pinpoint a novel therapeutic target that can effectively impact the TME, particularly in conjunction with ICIs to augment the efficacy of immunotherapy.

To identify immunotherapy resistance modulators, we performed a CRISPR cas9 screening in vivo and an RNA-sequencing for clinical immunotherapy resistance BC samples. We found that Enolase 1 (ENO1) as a potent regulator for PD-L1 blockade treatment. ENO1 is involved in multiple tumor-related biological processes, such as growth, metastasis, and resistance to treatment [8–10]. Zhu et al. found that ENO1 S249 glycosylation could enhance PD-L1 expression and inhibits T cell-mediated immunity against tumor cells. Blockade ENO1 synergizes with PD-L1 monoclonal antibody significantly promote antitumor immune response [11]. Our previous study found that ENO1 m6A modification promoted the proliferation of BC cells and was associated with the prognosis of BC patients [12]. However, its role in reshaping the tumor immune microenvironment and immunotherapy tolerance, particularly in BC, remains unclear. Therefore, we used BC cell lines, patient samples, public databases, and murine models to comprehensively explore the relevance between ENO1 and immune characteristics of BC and the efficacy of anti-PD-L1 therapy in BC.

## Materials and methods

### Clinical specimens

The mRNA expression profiles of BC patients were downloaded from The Cancer Genome Atlas Urothelial Bladder Carcinoma Collection (TCGA-BLCA) dataset (https://tcgadata.nci.nih.gov/tcga) and GEO dataset (GSE13507, https://www.ncbi.nlm.nih.gov/geo/). Our in-house BC cohort consisted of 96 BC patients who received radical BC surgery at the Affiliated Hospital of Qingdao University (Qingdao, Shandong, China) from January 2013 and December 2021. Our immunotherapy cohort consisted of 30 participants. We categorized BC patients into progressive disease (PD), stable disease (SD), partial response (PR), and complete response (CR) groups based on changes in tumor size after receiving immunotherapy. PD refers to an increase in tumor size or the emergence of new tumors. SD indicates no significant change in tumor size. PR refers to a reduction in tumor size, but not complete disappearance. CR refers to the complete disappearance of the tumor, with this state lasting for at least one month. Each participant provided written informed consent, and the research protocol received approval from the Ethics Committee of the Affiliated Hospital of Qingdao University.

### Cell lines

The mouse BC cell line MB49 and the human BC cell line T24 were acquired from the Cell Bank of the Chinese Academy of Sciences (Shanghai, China). Both cell lines were cultured in RPMI-1640 medium (Gibco, USA) supplemented with 10% fetal bovine serum (FBS) (Hyclone, USA) and 1% penicillin/streptomycin (Invitrogen, USA), and maintained at 37 °C in a humidified atmosphere containing 5% CO2. The MycoAlert Mycoplasma Detection Kit was used to evaluate the presence of mycoplasma contamination in the cells.

### Tumor models and treatments

MB49 (5 × 10^5^) cells were subcutaneously injected into the C57BL/6J mice flank. Tumor growth was monitored, and tumor volume was calculated using the formula: long diameter × short diameter^2^/2. Tumor volumes greater than 1500 mm^3^ were considered significant events in the survival experiments.

Mice were administered luciferase-labeled tumor cells for in vivo tumor imaging, and isoflurane inhalation was performed to induce anesthesia. Subsequently, the mice received an injected intraperitoneally of 150 mg/kg D-luciferin sodium salt (Absin, catalog no. 1103404-75-7), and imaged 10 min post-injection using an IVIS Spectrum In Vivo imaging system (PerkinElmer, USA).

For Immune checkpoint inhibitors treatment, mice bearing WT or ENO1-KO MB49 cells were intraperitoneally administered anti-PD-L1 mAbs (200 µg per mouse; BioXCell, catalog no. BE0101, clone: 10 F.9G2) or appropriate isotype control mAbs on days 7, 9, 11, 13, and 15. CD8^+^ T cells were depleted by injecting anti-mouse CD8 mAbs (200 µg per mouse; BioXCell, catalog no. BE0117, clone: YTS169.4) into the peritoneum. The injections were given on days − 6, − 3, and − 1 before tumor challenge, and the same dose was repeated on days 7, 9 and 11 after the tumor challenge. The orthotopic tumor model was established via surgical implantation. Mice were anesthetized with 2.5% isoflurane and injected with either fLuc-WT cells or fLuc-ENO1-KO cells (5 × 10^5^) into the bladder using insulin syringes. After several days of recovery, mice were randomized into different groups based on tumor burden and received regular administration. Anti-PD-L1 antibodies for animal experiments were administered via intraperitoneal injection at a dose of 200 µg per mouse. ENO1 inhibitor (ENOblock) or SPP1 inhibitor for animal experiments was administered via intraperitoneal injection at a dose of 10 mg/Kg per mouse.

### In vivo genome-wide CRISPR screen

The construction of a mouse pooled lentiviral library for CRISPR screening was purchase form GENECHEM company. There were 6 sgRNAs for each gene in the library. MB49 cells were infected with the lentivirus library with MOI of 0.4 for 48 h and selected by puromycin treatment (2 µg/ml) for 4 days. For in vivo screening, the transduced MB49 cells were subcutaneously transplanted to C57BL/6 mice and treated with anti-PD-L1 antibody or isotype control (three times per week) on day 6; tumors were harvested on day 20. The harvested tumors were subjected for genomic DNA extraction (DNA Blood Midi Kit, QIAGEN), sgRNA amplification, and NGS on an Illumina HiSeq to determine sgRNA abundance.

### shRNAs, siRNAs, CRISPR/Cas9 konckout, and plasmids

Short hairpin RNA (shRNA) constructs targeting ENO1 was purchased from GeneChem Co., Ltd. These constructions were used to establish cell lines with stable expression of the targeted genes via shRNAs. Cells were seeded into six-well plates at a density of 2 × 10^5^ cells/well. Following a 24-hour incubation, lentivirus particles were used for transfection. Afterward, the cells that were transfected were subjected to selection using puromycin at specific concentrations before use. Cells were transfected with siRNAs using established standard procedures. The cells were employed in experiments 48 h after transfection. ENO1 was knocked out using the CRISPR/Cas9 gene editing system, followed by puromycin selection. Transfection efficiency was evaluated via reverse transcription-quantitative polymerase chain reaction (RT-qPCR) and Western blot analysis. The overexpression plasmids His-SPP1 and control were commercially obtained from Huada company.

### Flow cytometry

Tumor samples were digested for 60 min at 37C with 25g/mL Hyaluronidase (Solarbio, H8030) and 160g /mL Collagenase (Solarbio, C8140). Single-cell suspensions were obtained through a 70-m cell strainer (MACS SmartStrainers, Miltenyi Biotec, catalog no. 130-098-462). These suspensions were then treated with Fc-block (Elabscience, catalog no. E-AB-F0997A) to inhibit nonspecific Fc-receptor-mediated binding before staining. Marker staining was performed using fixable viability dye (BD Pharmingen, catalog no. 565388), Percp/Cyanine 5.5-conjugated anti-mouse CD45 (Elabscience, E-AB-F1136J), APC-conjugated anti-mouse CD3 (Elabscience, E-AB-F1013E), or PE-conjugated anti-mouse CD8 (Elabscience, E-AB-F1104D), PE-conjugated anti-mouse GZMB (ThermoFisher Scientific, 12-8898-82),PE-conjugated anti-mouse IFN-(Elabscience, E-AB-F1101D), FITC-conjugated anti-mouse GZMB (ThermoFisher Scientific, 11-8898-82), FITC-conjugated anti-mouse IFN- (Elabscience, E-AB-F1101C), FITC Anti-Mouse/Human CD11b Antibody (Elabscience, E-AB-F1081C), PE anti-Mouse CD206/MMR antibody (Elabscience, E-AB-F1135D), or APC anti-Mouse CD86 antibody (Elabscience, E-AB-F0994E) on ice in the dark for 40min. Subsequently, the results were analyzed using a flow cytometer (CytoFLEX S; Beckman Coulter), with data processed using CytExpert software.

Tumor tissues are surgically extracted from mice, and non-target tissues are removed. The tumor tissue is cut into small pieces (1–2 mm³), washed with saline to remove blood, and placed into brown sample storage tubes containing MACS^®^ Tissue Storage Solution (Miltenyi Biotec, 130-100-008). The samples are transported to the laboratory at 4 °C and processed for tissue dissociation within 1 h.

### scRNA-seq

The tissue is removed from the storage tubes and washed in chilled PBS. The tissue is further cut into pieces of 2–3 mm and processed into a single-cell suspension using the Tumor Dissociation Kit, human (Miltenyi Biotec, 130-096-730). Red blood cells are lysed using Red Blood Cell Lysis Solution (Miltenyi Biotec, 130-094-183). The cells are resuspended in PBS + 0.04% BSA + RNA inhibitor (1U/µL). Cell viability, concentration, and aggregation rate are assessed using the LUNA-FL™ Automated Fluorescence Cell Counter (Cat# L20001, Logos Biosystems) with AOPI dye (Cat# F23001, Logos Biosystems), adjusting the cell concentration to 700–1200 cells/µL.Chromium Next GEM Single Cell 3’ Reagent Kits v3.1 (Dual Index) are used for each sample, loading 16,000 cells to generate water-in-oil droplets. Single-cell cDNA libraries are amplified using PCR to obtain sufficient template for sequencing. The amplified cDNA is processed for library construction according to Illumina sequencing platform requirements, including end repair, A-tailing, and adapter ligation.Library quality and concentration are assessed using the Qubit™ 1X dsDNA Assay Kit, high sensitivity (Qubit 4.0), the StepOnePlus™ Real-Time PCR System for molar concentration, and LabChip Touch for insert size analysis. Sequencing is performed on the Illumina NovaSeq6000 platform with PE150 read length. FastQC is used for sequencing data quality control, removing low-quality sequences and adapter contamination to generate high-quality data. Cellranger 6.0.1 is used to generate matrix files from raw data. Seurat is employed for quantification of cell expression and clustering analysis, with dimensionality reduction techniques like tSNE or UMAP used to visualize cell-type distribution. Cell annotation is conducted using SingleR, pseudotime analysis with Monocle 2.0, and GO enrichment and KEGG analysis with clusterProfiler. Cell-cell ligand-receptor interactions are analyzed using tools CellChat.

### Differentiation and polarization of BMDMs

A cell suspension was obtained by harvesting bone marrow from the femur and tibia of ENO1-WT and ENO1-KO mice and filtering it through a 70-µm cell strainer. Following centrifugation and lysis of red blood cells, the resulting bone marrow cells were cultured in RPMI-1640 medium (Gibco) supplemented with 10% FBS (Gibco), 1% penicillin/streptomycin (Gibco), and 50 µM 2-mercaptoethanol (Sigma), along with 20 ng/mL of macrophage colony-stimulating factor (M-CSF; PeproTech) for 5–7 days. Subsequently, the bone marrow-derived macrophages (BMDMs) were polarized into M1-like (antitumoral-type) macrophages through stimulation with 100 ng/mL of LPS (Sigma) and 20 ng/mL of IFN-γ (PeproTech) for 24 h.

### T cell culture and T-cell-mediated tumor cell killing assay

Human T cells were isolated from peripheral blood samples, and mouse T cells were obtained from spleens tissue samples. Peripheral blood mononuclear cells were isolated using Ficoll density gradient centrifugation, CD8^+^ T cells were then enriched using the MagCellect Human CD8^+^ T Cell Isolation Kit (R&D Systems) and the MagCellect Mouse CD8^+^ T Cell Isolation Kit (R&D Systems) following the instructions provided by the manufacturer. Human CD8^+^ T cells were cultured in RPMI-1640 supplemented with 10% FBS, 100 U/mL penicillin and streptomycin, 100 IU/mL recombinant human IL2, 2 mg/mL anti-human CD3 antibody, and 1 mg/mL anti-human CD28 antibody. Mouse CD8^+^ T cells were cultured in a complete RPMI-1640 medium with 2 mg/mL anti-mouse CD3 antibody and 1 mg/mL anti-mouse CD28 antibody. Following 5 days of stimulation, CD8^+^ T cells were collected and cocultured with different target cells.

### Analysis of in vitro T-cell proliferation and activation

To assess the functionality of CD8^+^ T cell post-treatment, the cells were initially collected and then treated with 4% paraformaldehyde at 4 °C for 1 h. Following fixation, the cells were washed twice with PBS and resuspended in 500 mL of Triton X-100 for 15 min for permeabilization. Next, the cells were rewashed again and incubated with antibodies against Ki67, IFN-γ, and GZMB for 30 min at 4 °C. Fluorescence intensity was then analyzed using flow cytometry.

### Measurements of the stability of mRNA

To assess the stability of SPP1 mRNA, cells were treated with ActD (5 µg/mL) for the specified time. Following treatment, SPP1 mRNA expression levels were evaluated by RT-qPCR.

### Co-Immunoprecipitation (Co-IP)

Cells were cultured in 10 cm dishes, followed by scraping and lysing in IP buffer (containing 20 mM Tris-HCl, pH 7.5, 150 mM NaCl, 1 mM Na2EDTA, 1 mM EGTA, 1% Triton X-100, 2.5 mM sodium pyrophosphate, 1 mM beta-glycerophosphate, 1 mM Na3VO4, 1 µg/ml leupeptin, and 1 mM PMSF) on ice for 30 min. After centrifugation at 13,000 × g for 15 min, the supernatants were incubated with protein A/G beads conjugated with the specified antibodies for 5 h at 4 °C. Immunocomplexes were then analyzed via western blot using the specific antibodies.

### Real-time quantitative PCR analysis (RT-qPCR)

Total RNA was extracted using TRIzol reagent (Invitrogen, USA) following the manufacturer’s instructions. The extracted RNA was reverse transcribed into cDNA using the PrimeScript™ RT reagent kit (Perfect Real Time) (Takara, Japan). RT-qPCR was performed using the Roche LightCycler 480II real-time PCR detection system (Roche, Basel, Switzerland). Relative mRNA expression levels were determined using the 2^−ΔΔCt^ method with GAPDH serving as the internal normalization control.

### Western blot

Western blot analysis was performed following previously established protocols [13]. Antibodies from various sources were utilized, including ENO1 (Proteintech, #11204-1-AP, 1:1000), His (Proteintech, 66005-1-Ig, 1:1000), ITGA4 (Proteintech, 19676-1-AP, 1:1000), ITGB1 (Proteintech, 12594-1-AP, 1:1000) and GAPDH (Proteintech, #10494-1-AP, 1:5000). These antibodies were incubated overnight at 4 °C. Next, the membranes were treated with HRP-conjugated secondary antibodies (Jackson ImmunoResearch, #309-005-003, 1:10,000) for 1 h at room temperature. Immunoreactivity was visualized using chemiluminescence following the manufacturer’s guidelines.

### Immunohistochemistry (IHC)

The IHC staining was employed to assess the expression levels of ENO1, SPP1, CD206, CD86, Ki67, and CD8A in both BC patients and mouse tumor tissues, as described in the previous study [14].

### Statistical analysis

Statistical differences between groups were assessed using two-tailed Student’s *t*-test, one-way ANOVA, and two-way ANOVA. The Kaplan–Meier survival curves were generated and analyzed using the logrank (Mantel–Cox) test with GraphPad Prism Software in all mouse-related experiments. The significance level in the figure legend is denoted as follows: *p < 0.05, **p < 0.01, ***p < 0.001, and ****p < 0.0001 (where *p < 0.05 was considered statistically significant).

## Results

### In vivo CRISPR-cas9 screening identifies ENO1 as an immunotherapy resistance modulator in BC

**Fig. 1:**
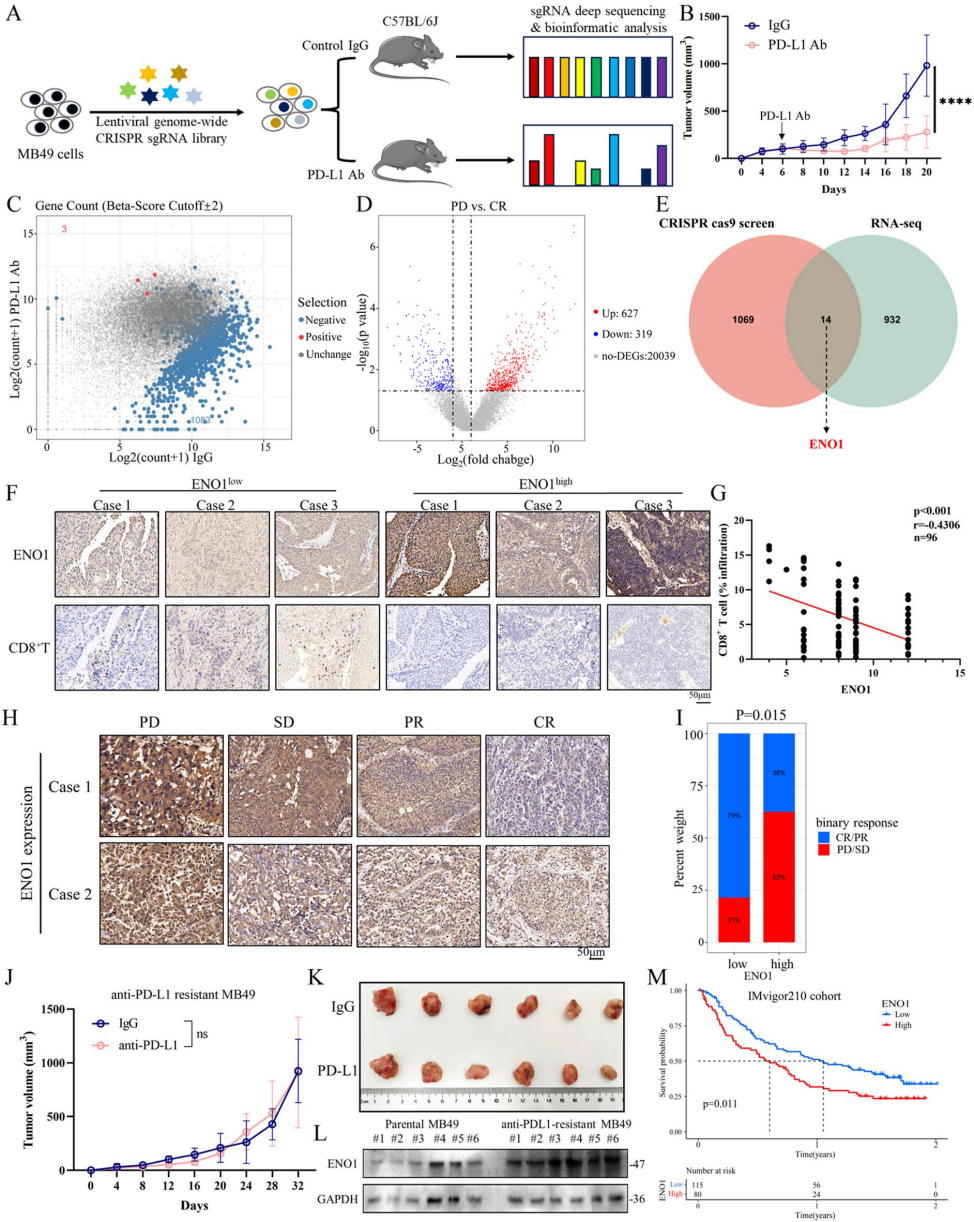
In vivo CRISPR-cas9 screening identifies ENO1 as an immunotherapy resistance modulator in BC. (A) Schematic of the in vivo genome-wide CRISPR cas9 screening to identify genes associated with anti-PD-L1 responsiveness. **(B)** The in vivo efficacy of anti-PD-L1 antibody in C57BL/6J mice with xenografts of MB49 cells. The mice were treated with control IgG or anti-PD-L1 antibody for 14 days, and average tumor sizes for each group are plotted. **(C)** The relative enrichment of genes in the genome-wide CRISPR cas9 screening for anti-PD-L1 responsiveness. Total 1083 genes were depleted in anti-PD-L1-treated tumors (Beta-score <-2). **(D)** Volcano plot of RNA-seq for progressive disease (PD) or complete response (CR) human BC tumor tissues (*n* = 3 biologically independent samples per group). Differentially expressed genes were identified with the threshold of |log2(fold change)| >1 and false discovery rate (FDR) < 0.05. **(E)** On integrating the CRISPR cas9 screening and RNA-seq results, 14 genes (ENO1, ANLN, CCL4, CDK1, FABP3, KLRC1, LAMP3, NUAK2, PTPRCAP, PTPRZ1, RORC, SGPL1, TMEM141, USP2) were finally identified. **(G)** Representative images of IHC staining for ENO1 and CD8^+^T in human BC samples. Scale bars: 50 μm **(H)** Correlation between protein levels of ENO1 and CD8^+^ T cell infiltration was determined by IHC staining (*P* < 0.001, *r*=-0.4306, *n* = 96). **(I)** Representative images of IHC staining for ENO1 in progressive disease (PD), stable disease (SD), partial response (PR), and complete response (CR) samples. **(J)** Bar plot illustrating the response rates of anti-PD-L1 therapy. Blue bars represent CR/PR, Red bars represent PD/SD. Scale bars: 50 μm Two-tailed χ^2^ test. **(K**,** L)** Tumor growth curves and tumor sizes of anti-PD-L1 resistant MB49 cells (*n* = 6 per group). Two-side unpaired Student’s t-test. Data are presented as mean ± SD. ns, no significant. **(M)** Western blotting demonstrating ENO1 expression in anti-PD-L1 resistance and parental MB49 cells. **(N)** Kaplan-Meier overall survival (OS) curve for 195 BC patients in the IMvigor210 cohort stratified by high or low ENO1 expression. Two-sided log-rank test

To systemically evaluate cell-intrinsic regulators of immunotherapy resistance, we performed a pooled loss-of-function genetic screen using immunocompetent C57BL/6 mice (Fig. 1A). We transduced MB49 cells with a library of lentivirus encoding Cas9 and 130,209 sgRNAs targeting 21,786 genes, and transplanted MB49 cells into mice and then treated with anti-PD-L1 antibody. After 2 weeks, tumors were harvested for amplicon sequencing (Fig. 1B). The results found that total 1083 genes were depleted in anti-PD-L1-treated tumors (Beta-score <-2), suggesting that loss-of-function of these genes increased sensitivity to anti-PD-L1 (Fig. 1C). To further identify genes, we performed RNA-seq on 6 human BC tissues with different response rates to anti-PD-L1 therapy (CR, 3; PD, 3). Compared with CR, 627 genes were upregulated and 319 genes were downregulated in PD tissues (Fig. 1D). On integrating the CRISPR screen and RNA-seq results, 14 genes (ENO1, ANLN, CCL4, CDK1, FABP3, KLRC1, LAMP3, NUAK2, PTPRCAP, PTPRZ1, RORC, SGPL1, TMEM141, USP2) were finally identified (Fig. 1E). Among these genes, we focus on ENO1 that was highly expressed in BC and was closely related to the prognosis of BC patients [12]. However, the role of ENO1 in the immune evasion of BC is unclear.

Analysis of the TCGA-BLCA dataset revealed a positive association between ENO1 expression and exhausted T cell-related markers (PDCD1, HAVCR2, LAG3, CXCL3) (Additional file1A). Additional confirming using IHC staining of BC tissues revealed a correlation between increased expression of ENO1 and decreased infiltration of CD8^+^ T cells (*p* < 0.001, *r*= -0.4306, *n* = 90) (Fig. 1F, G), indicating its potential involvement in regulating immune evasion in BC. The analysis of the correlation between ENO1 expression and immunotherapy response demonstrated elevated ENO1 expression in patients with progressive disease/stable disease (PD/SD) in contrast to those with partial response/complete response (PR/CR) (Fig. 1H). Patients in the ENO1-high group exhibited a 38% clinical response rate, whereas those in the ENO1-low group had a 79% response rate (Fig. 1I). Further validation was conducted using an in vivo mouse tumor model resistant to anti-PD-L1 therapy. By employing the MB49 BC model and performing serial in vivo and in vitro passaging while using an anti-PD-L1 antibody, anti-PD-L1-resistant MB49 cell lines were derived after seven cycles of passaging, displaying unresponsiveness to anti-PD-L1 treatment in vivo (Fig. 1J, K). To further validate the resistant phenotype, anti-PD-L1-resistant MB49 cells were co-cultured with CD8^+^ T cells in the presence of anti-PD-L1 antibody. Compared with parental MB49 cells, PD-L1-resistant MB49 cells exhibited significantly reduced susceptibility to CD8^+^ T cell-mediated killing under anti-PD-L1 treatment (Additional file1B, C). Western blot analysis revealed significant upregulation of ENO1 in resistant MB49 tumors (Fig. 1L). In the IMvigor210 cohort, there was a correlation between high ENO1 expression and poor prognosis in patients with BC (Fig. 1M). The IMvigor210 cohort a single-arm phase II research that examined the use of atezolizumab in treating metastatic urothelial carcinoma. These results suggest that ENO1 is significantly associated with impaired antitumor immunity and resistance to immunotherapy.

### Tumor-Intrinsic ENO1 deficiency inhibits tumorigenesis and triggers antitumor immunity

**Fig. 2:**
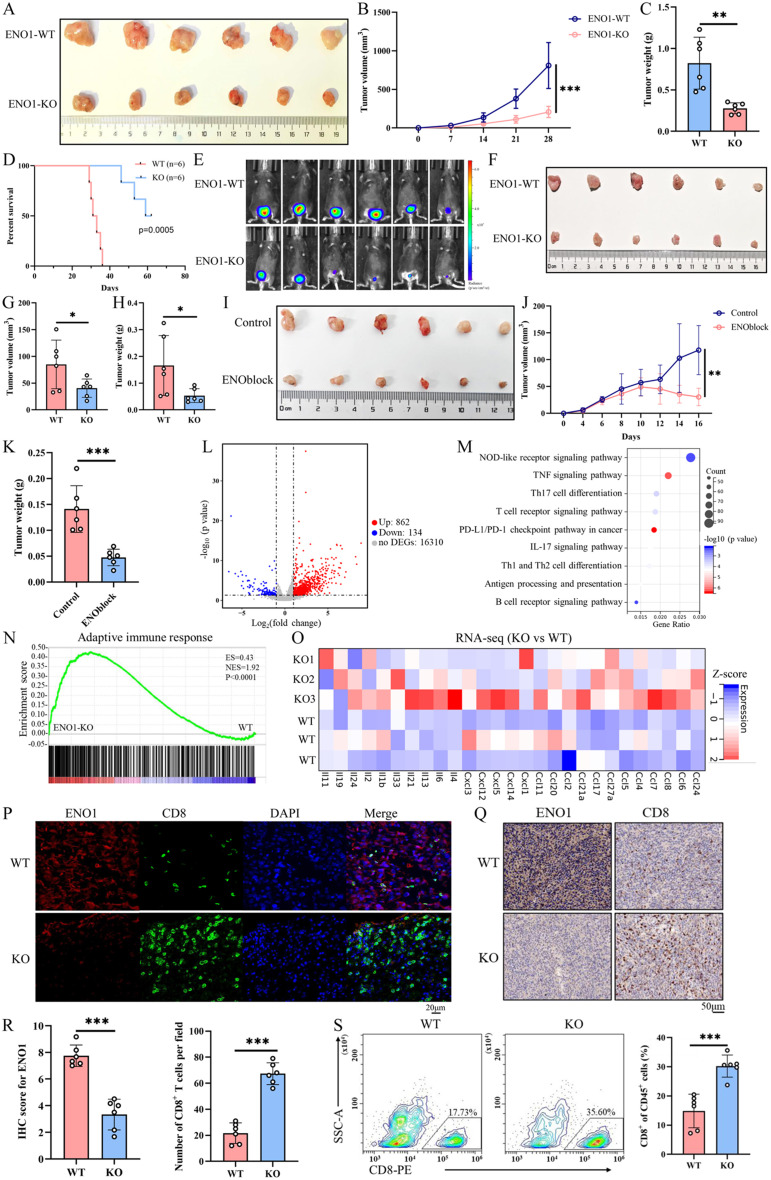
Tumor-intrinsic ENO1 deficiency inhibits tumorigenesis and triggers antitumor immunity. (A-C) Subcutaneous inoculation of WT or ENO1-KO cells into C57BL/6J mice (*n* = 6 per group) followed by measurement of tumor sizes (**A**), volumes (**B**), and weights (**C**). Two-side unpaired Student’s t-test. Data are presented as mean ± SD. **(D)** Kaplan-Meier survival curves of C57BL/6J mice inoculated with WT or ENO1-KO MB49 cells. Tumor volumes exceeding 1500mm^3^ were considered events (*n* = 6 per group). Two-sided log-rank test. **(E-H)** In vivo bioluminescence imaging of a bladder orthotopic tumor model established by subcutaneously inoculating luciferase-labeled WT or ENO1 MB49 cells into the bladder of C57BL/6J mice (*n* = 6 per group). The signals were measured on 20 days using an IVIS Spectrum In Vivo imaging system (**E**), and tumor sizes (**F**), volumes (**G)**, and weight (**H**) were measured. Two-side unpaired Student’s t-test. Data are presented as mean ± SD. **(I-K)** Subcutaneous inoculation of MB49 cells into C57BL/6J mice followed by treatment with 8 mg/kg ENOblock every other day (*n* = 6 per group). Tumor sizes (**I**), volumes (**J**), and weight (K) were measured. Two-side unpaired Student’s t-test. Data are presented as mean ± SD. **(L)** Volcano plot of RNA-seq data for WT or ENO1-KO tumors inoculated into C57BL/6J mice (*n* = 3 biologically independent samples per group). Differentially expressed genes were identified with the threshold of |log2(fold change)| >1 and false discovery rate (FDR) < 0.05. **(M)** KEGG analysis for differentially expressed genes to show immune-related pathways. **(N)** GSEA revealing immune-associated pathways correlated with ENO1 expression in the RNA-seq dataset. **(O)** Heatmap of RNA-seq data for interleukin and chemokine family member expression. **(P-R)** Representative images of IHC and mIHC staining for ENO1 and CD8^+^ T cells in WT or ENO1-KO tumor tissues (*n* = 6 per group). Expression levels of the indicated proteins displayed. Two-side unpaired Student’s t-test. Data are presented as mean ± SD. **(S)** Flow cytometric analysis of tumor-infiltrating CD8^+^ T cells in WT or ENO1-KO tumor tissues (*n* = 6 per group). Two-side unpaired Student’s t-test. Data are presented as mean ± SD. *p < 0.05, **p < 0.01, ***p < 0.001

To elucidate the role of ENO1 in regulating immune evasion within the tumor microenvironment, ENO1 expression levels across different cell types were analyzed using BC single-cell RNA sequencing (scRNA-seq) data [15]. The result showed that ENO1 was highly expressed and differentially expressed in tumor cells, suggesting that tumor-intrinsic ENO1 may contribute to the immune evasion of BC (Additional file2A-D). To investigate the biological roles of tumor-intrinsic ENO1 in the development of tumors immune evasion, the CRISPR-Cas9 technology was utilized to specifically knockout the expression of ENO1 gene in the MB49 cell line. RT-qPCR and western blot analyses confirmed significant downregulation of ENO1 (Additional file2E, F). Subsequent subcutaneous injection or orthotopic tumor model of immunocompetent mice with WT or ENO1-KO cells demonstrated that the absence of ENO1 in the tumor cells significantly inhibited tumorigenesis, as evidenced by reduced tumor volume, weight, as well as the survival rate in the ENO1-KO group (Fig. 2A-H). Additionally, ENO1 inhibitor, ENOblock significantly reducing tumor volume and weight (Fig. 2I-K). Similar results were validated in lung metastasis models as well (Additional file2G-P).

To investigate whether inhibition of ENO1 enhanced antitumor immunity, transcriptome analysis was initially conducted to screen for differential biological processes and pathways between WT and ENO1-KO tumor tissues. In total, we identified 996 differentially expressed genes including 862 upregulated genes and 134 downregulated genes (Fig. 2L). Among the DEGs, genes encoding cytotoxic molecules (e.g., GZMB, GZMA, and IFN-γ) exhibited enhanced expression. Consistently, pathway enrichment analysis showed that ENO1 expression was associated with several immune-related pathways in BC, such as antigen processing and presentation, NOD-like receptor signaling pathway, TNF signaling pathway, Th17 cell differentiation, T cell receptor signaling pathway, and so on (Fig. 2M). Similarly, gene set differentially enriched in the low ENO1 expression group were related to adaptive immune response, positive regulation of leukocyte mediated immunity, myeloid cell activation involved in immune response, Type 2 immune response, and chemokine activity, all of which mainly stimulate antitumor immunity (Fig. 2N, Additional file2Q). Next, we analyzed cytokine expression based on the observation that the chemokine activity was activated in ENO1-KO tumors. A heatmap of RNA-seq showed that most of interleukin family and chemokine family were upregulated in ENO1-KO tumor tissues (Fig. 2O). Furthermore, increased levels of tumor-infiltrating CD8^+^ T cells were demonstrated in ENO1-KO tumors through IHC staining, mIHC, and flow cytometric analysis (Fig. 2P-S). These results indicated that tumor-intrinsic ENO1 deficiency triggers antitumor immunity in BC.

### Single-cell RNA sequencing reveals the difference of CD8^+^ T cell subgroups of ENO1-WT versus ENO1-KO tumors

**Fig. 3:**
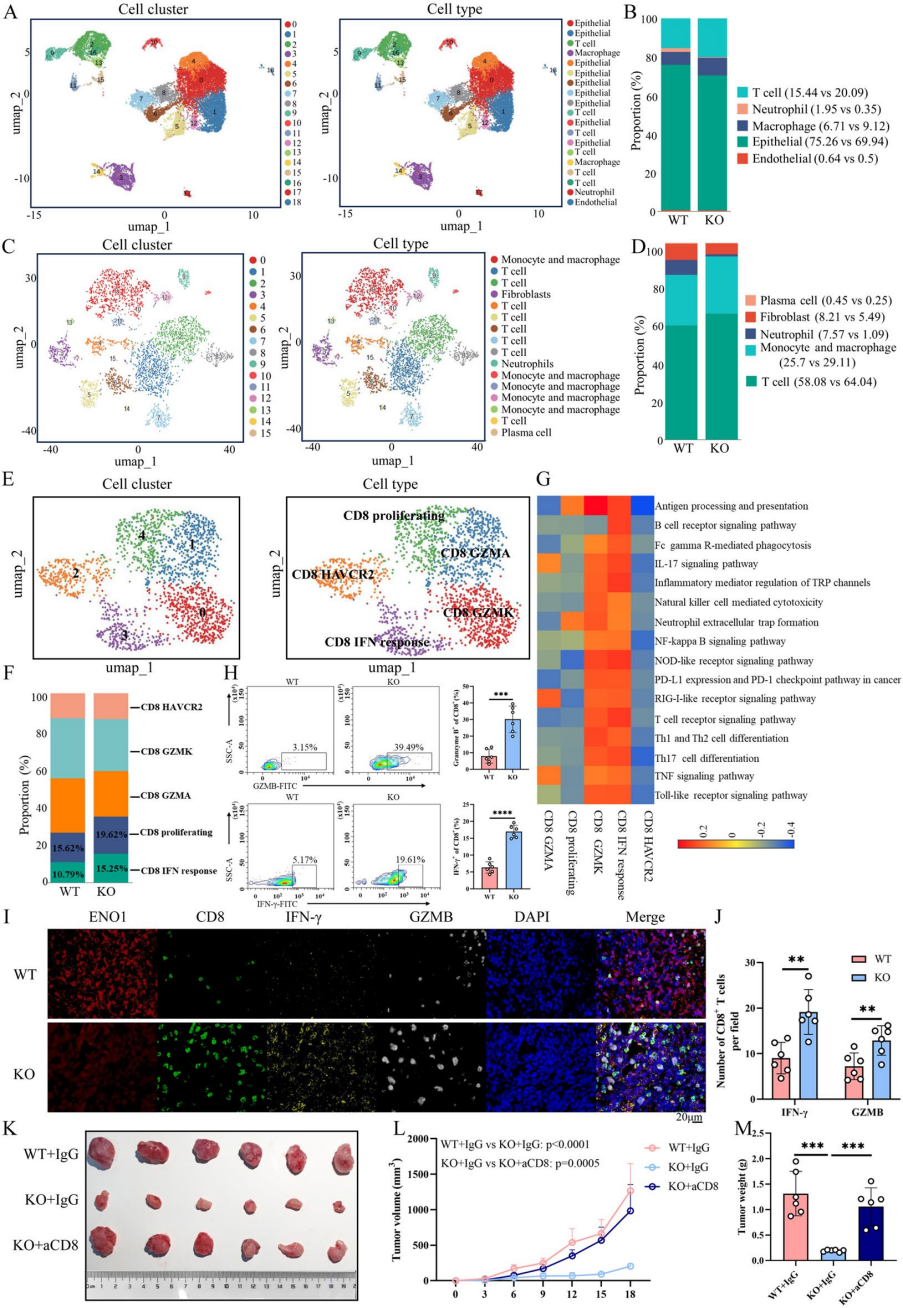
Single-cell RNA sequencing reveals the difference of CD8^**+**^ T cell subgroups of ENO1-WT versus ENO1-KO tumors. **(A)** The umap plot displaying all cells isolated from tumor tissues from the WT and KO groups, with each color representing a distinct cell cluster and cell type. **(B)** Bar plot showing the proportion of each cell subpopulation in the WT and KO groups. **(C)** The t-SNE plot of CD45^+^ cells subpopulations, color coded by cell cluster and cell type. **(D)** Bar plot showing the proportion of each CD45^+^ cells subpopulations subpopulation in the WT and KO groups. **(E)** The umap plot of CD8^+^ T cells subpopulations, color coded by cell cluster and cell type. **(F)** Bar plot showing the proportion of each CD8^+^ T-cell clusters. **(G)** Heatmap of differentially activated pathway among all the CD8^+^ T-cell clusters. **(H**,** I)** Flow cytometric analysis and mIHC of tumor-infiltrating IFN-γ^+^ or GZMB^+^ CD8^+^ T cells in WT or ENO1-KO tumor tissues (*n* = 6 per group). Two-side unpaired Student’s t-test. Data are presented as mean ± SD. **(K-M)** Isotype control (IgG) or anti-mouse CD8 antibody administered on days − 6, − 3, and − 1 before tumor challenge, with the same dose repeated on days 7, 9 and 11 after tumor challenge. Tumor sizes (**K**), volumes (**L)**, and weight (**M**) were measured. Two-way ANOVA with Tukey’s multiple comparison test. Data are presented as mean values ± SD. ***p < 0.001, ****p < 0.0001

To uncover the heterogeneity in the immune microenvironment at single-cell resolution, single-cell RNA sequencing (scRNA-seq) on both the WT and ENO1-KO tumors was performed. The clustering analysis was performed to identify different cell types within the tumor microenvironment. Uniform Manifold Approximation and Projection (UMAP) analysis of the total cell populations identified 18 subclusters comprising endothelial cells, epithelial cells, monocytes/macrophages, neutrophils, and T cells (Fig. 3A). The abundance of cancer cells decreased, while T cell abundance increased in ENO1-KO tumors (Fig. 3B). A subsequent tumor-infiltrating CD45^+^ immune cells analysis identified 15 clusters comprising T cells, monocytes/macrophages, plasma cells, neutrophils, and fibroblasts (Fig. 3C). T cells were enriched in ENO1-KO tumors, whereas neutrophils showed a higher infiltration rate in WT tumors (Fig. 3D).

In the CD8^+^ T cell subgroup analysis, proliferating CD8^+^ T cells, GZMK^+^ CD8^+^ T cells, naive CD8^+^ T cells, HAVCR2^+^ CD8^+^ T cells, and IFN-responsive CD8^+^ T cells were identified as distinct subpopulations based on known markers (Fig. 3E). ENO1-KO tumors exhibited higher infiltrating CD8^+^ T cells, predominantly comprising proliferating CD8^+^ T cells and IFN-responsive CD8^+^ T cells (Fig. 3F). The KEGG enrichment analysis revealed an increase in immune-associated pathways (such as PD-L1 expression and PD-1 checkpoint pathway in cancer, Th17 cell differentiation, Th1 and Th2 cell differentiation, T cell receptor signaling pathway, and Toll-like signaling pathway) in these CD8^+^ T cell subpopulations (Fig. 3G). Flow cytometry and mIHC corroborated the enhanced production of IFN-γ and GZMB in tumor-infiltrating CD8^+^ T cells from ENO1-KO samples (Fig. 3H-J). To investigate the vital function of CD8^+^ T cells in the immune response to tumors, in vivo knockdown experiments were performed by utilizing anti-mouse CD8 monoclonal antibodies were conducted. The depletion of CD8^+^ T cells significantly improved the growth of ENO1-KO tumors (Fig. 3K-M), highlighting the contribution of tumor-intrinsic ENO1 deficiency in promoting antitumor immunity via modulation of CD8^+^ T cell infiltration levels in the tumor microenvironment.

### Tumor intrinsic ENO1 inhibits the function of CD8^+^ T cells via the SPP1-ITGA4/ITGB1 pathway

**Fig. 4:**
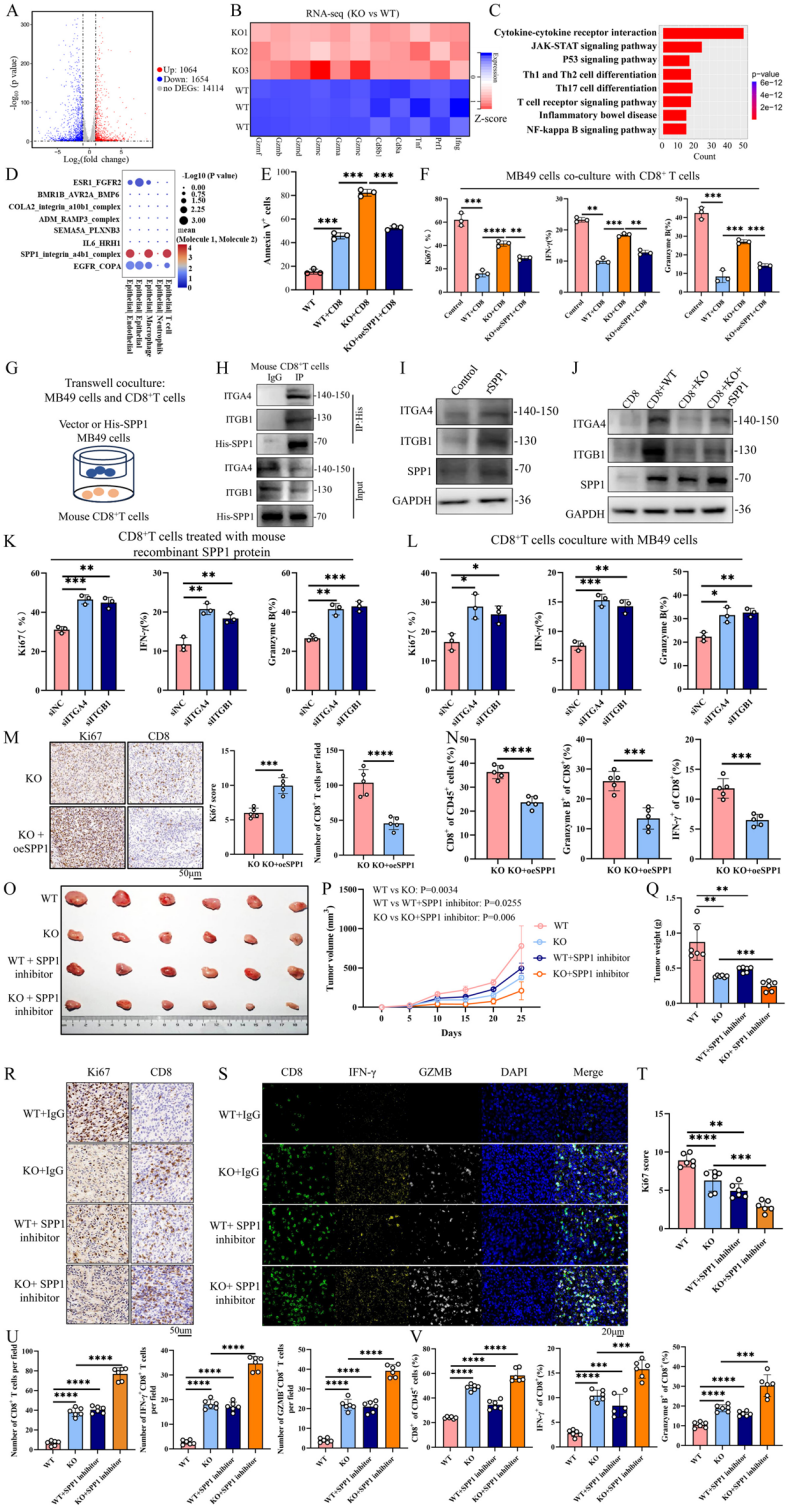
Tumor intrinsic ENO1 inhibits the function of CD8^**+**^ T cells via SPP1. **(A)** Volcano plot of RNA-seq data for CD8^+^ T cells that cocultured with ENO1-WT or ENO1-KO MB49 cells (*n* = 3 biologically independent samples per group). Differentially expressed genes were identified with the threshold of |log2(fold change)| >1 and false discovery rate (FDR) < 0.05. **(B)** Genes encoding cytotoxic molecules (e.g., PRF1, TNF, CD8A, CD8B1, GZMA, GZMB, GZMC, GZMD, GZME, and GZMF) expression levels in CD8^+^ T cells. **(C)** KEGG enrichment analysis for differentially expressed genes to show immune-related pathways. **(D)** CellChat to was used to delineate cell-cell communications in the single-cell RNA sequencing dataset. **(E)** Apoptosis assay results of WT, ENO-KO, or ENO1-KO + oeSPP1 cells in the presence of mouse CD8^+^ T cells. *n* = 3 biologically independent samples per group. One-way ANOVA with Dunnett’s multiple comparisons test. Data are presented as mean values ± SD. **(F)** Flow cytometry analysis showed Ki67, IFN-γ, GZMB expression of mouse CD8^+^ T cells that were cocultured with WT, ENO-KO, ENO1-KO + oeSPP1 cells. *n* = 3 biologically independent samples per group. One-way ANOVA with Dunnett’s multiple comparisons test. Data are presented as mean values ± SD. **(G)** A schematic representation of a transwell coculture assay involving mouse CD8^+^ T cells combined with His-SPP1 MB49 cells. **(H)** Co-immunoprecipitation assays of CD8^+^ T cells were performed using anti-His antibody. *n* = 3 biologically independent samples per group. **(I)** Western blotting assay detected ITGA4 and ITGB1 protein expression in mouse CD8^+^ T cells with or without recombinant mouse SPP1 protein treatment. *n* = 3 biologically independent samples per group. **(J)** Western blotting assay detected ITGA4 and ITGB1 protein expression in mouse CD8^+^ T cells cocultured with WT or ENO1-KO cells, with or without recombinant mouse SPP1 protein. *n* = 3 biologically independent samples per group. **(K)** Flow cytometric analysis of Ki67, IFN-γ and GZMB expression in siITGA4 or siITGB1 CD8^+^ T cells treated with recombinant mouse SPP1 protein. *n* = 3 biologically independent samples per group. **(L)** Flow cytometric analysis of Ki67, IFN-γ and GZMB expression in siITGA4 or siITGB1 CD8^+^ T cells cocultured with MB49 cells. *n* = 3 biologically independent samples per group. Two-side unpaired Student’s t-test. Data are presented as mean ± SD. **(M)** IHC staining of Ki67 or CD8 expression in ENO1-KO or ENO1-KO + oeSPP1 tumors (*n* = 5 per group). Two-side unpaired Student’s t-test. Data are presented as mean ± SD. **(N)** Flow cytometric analysis of tumor-infiltrating CD8^+^ T cells, IFN-γ^+^ or GZMB^+^ CD8^+^ T cells in ENO1-KO or ENO1-KO + oeSPP1 tumor tissues (*n* = 5 per group). Two-side unpaired Student’s t-test. Data are presented as mean ± SD. **(O-Q)** C57BL/6J mice (*n* = 6 per group) were subcutaneously inoculated with WT or ENO1-KO cells. Mice received intraperitoneally treated with 10 mg/Kg SPP1 inhibitor every others day after tumor inoculation. Tumor sizes (**O**), volumes (**P**), and weight (**Q**) were measured. Two-way ANOVA with Tukey’s multiple comparison test. Data are presented as mean values ± SD. **(R-U)** Representative images of IHC and mIHC staining for Ki67, CD8, IFN-γ, GZMB in different tumor tissues (*n* = 6 per group). Expression levels of the indicated proteins displayed. Two-way ANOVA with Tukey’s multiple comparison test. Data are presented as mean ± SD. **(V)** Flow cytometric analysis of tumor-infiltrating CD8^+^ T cells, IFN-γ^+^ or GZMB^+^ CD8^+^ T cells in distinct tumor tissues (*n* = 6 per group). Two-way ANOVA with Tukey’s multiple comparison test. Data are presented as mean ± SD. *p < 0.05, **p < 0.01, ***p < 0.001, ****p < 0.0001

To investigate the mechanism by which ENO1 regulates T cell infiltration and function within the TME, human CD8^+^ T cells from peripheral blood and mouse CD8^+^ T cells from splenocytes were isolated and stimulated for coculture with WT or ENO1-KO cells (Additional file3A). Subsequently, cocultivation experiments were performed to evaluate the impact of ENO1 inhibition on T-cell responses. RNA-seq of CD8^+^ T cells identified 2718 differentially expressed genes including 1064 upregulated genes and 1654 downregulated genes (Fig. 4A). Among the DEGs, genes encoding cytotoxic molecules (e.g., PRF1, TNF, CD8A, CD8B1, GZMA, GZMB, GZMC, GZMD, GZME, and GZMF) exhibited enhanced expression (Fig. 4B). The functional enrichment analysis indicated that the enrichment for cytokine-cytokine receptor interaction was significantly enriched in CD8^+^ T cells (Fig. 4C). CellChat was used to map cell-cell communications in the scRNA-seq dataset. CellChat analyzed the differential increase of ligand-receptor pairings in tumors, distinguishing cancer cell as “sender cells” that expressed genes encoding secreted ligands and noncancer cells in the TME as “recipient cells” that expressed receptors. The results revealed that bladder cancer cells secrete SPP1, which may interact with the integrin α4/β1 (ITGA4/ITGB1) complex of T cells to influence T cell function (Fig. 4D). The cocultivation results revealed that ENO1 inhibition in BC cells significantly enhanced the killing ability of CD8^+^ T cells, while overexpression of SPP1 in ENO1-KO cells partially rescued this effect (Fig. 4E, Additional file3B-D). Moreover, cocultivation with ENO1-KO cells significantly increased the proportion of activated CD8^+^ T cells, as indicated by Ki67, IFN-γ, and GZMB expression. In contrast, overexpression of SPP1 in ENO1-KO cells or addition of recombinant SPP1 protein in the coculture system reduced the proportion of activated CD8^+^T cells (Fig. 4F, Additional file3E-G). The ELISA assay demonstrated that knockdown of ENO1 could decrease the SPP1 secretion of cancer cells, while overexpression of SPP1 in ENO1-KO cells could rescue it (Additional file3H, I). Subsequently, we determine whether the tumor cells-secreted SPP1 could influence CD8^+^T cells function by regulating ITGA4/ITGB1 expression. co-IP assays demonstrated that the His-tagged SPP1 secreted by BC cells can interact with the ITGA4/ITGB1 in M1-polarized BMDMs or THP-1 cells (Fig. 4G, H, Additional file3J, K). Moreover, recombinant SPP1 protein could enhanced ITGA4/ITGB1 expression in CD8^+^T cells (Fig. 4I, Additional file3L). In the coculture experiments, recombinant SPP1 protein could rescue ITGA4/ITGB1 expression in CD8^+^T cells that cocultured with ENO1-KO or shENO1 cells (Fig. 4J, Additional file3M). The flow cytometry analysis showed that knockdown of ITGA4 or ITGB1 can inhibit the effect of recombinant SPP1 protein or cancer cells on the function of CD8^+^T cells (Fig. 4K, L, Additional file3N, O). These results revealed that tumor-intrinsic ENO1 modulating CD8^+^T cells function through the SPP1- ITGA4/ITGB1 signaling pathway in the TME.

In vivo experiments demonstrated that overexpression of SPP1 in ENO1-KO cells increased tumor volume and weight compared to ENO1-KO tumors (Additional file3P-R) and reduced the abundance of CD8^+^ T cells, IFN-γ^+^ CD8^+^ T cells, and GZMB^+^ CD8^+^ T cells (Fig. 4M, N). Furthermore, Mice injected with WT and ENO1-KO cells were treated with SPP1 inhibitor, revealing that ENO1 knockdown combined with SPP1 inhibitor treatment significantly inhibited tumor growth (Fig. 4O-Q). IHC staining, mIHC, and the flow cytometry analysis showed a substantial increase in functional tumor-infiltrating CD8^+^ T cells, including IFN-γ^+^ CD8^+^ T cells and GZMB^+^ CD8^+^ T cells in the TME (Fig. 4R-V). These results suggest that tumor-intrinsic ENO1 inhibited the infiltration and function of CD8^+^ T cells in the TME via the SPP1 signaling pathway.

### ENO1 promotes the polarization of M2 TAMs via the SPP1-ITGA4/ITGB1 pathway

**Fig. 5:**
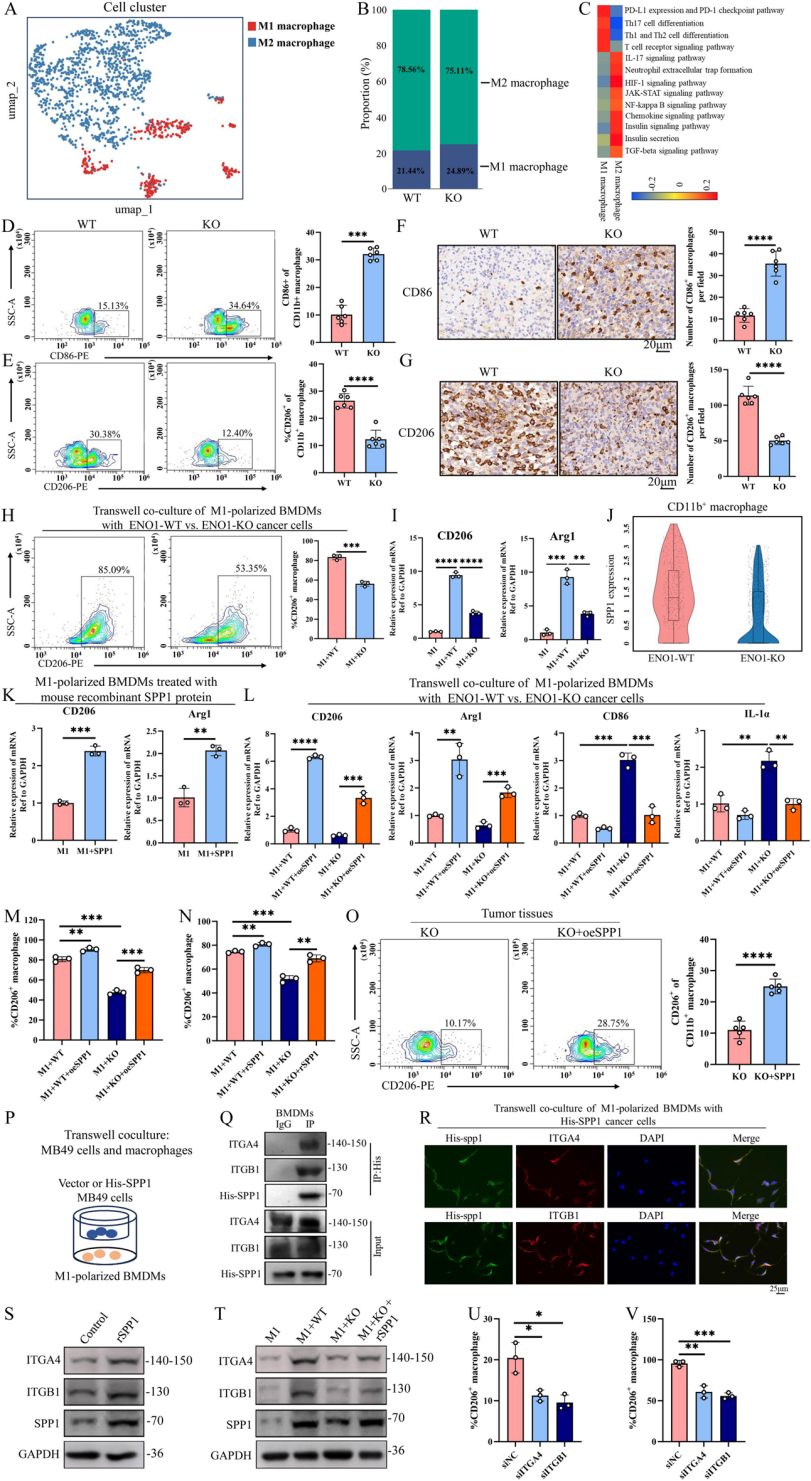
ENO1 promotes the polarization of M2 TAMs via SPP1 in TME. **(A)** The umap plot of M1 (antitumoral) and M2 (protumoral) macrophages from scRNA-seq data. **(B)** Bar plot presenting the proportion of M1 and M2 macrophages subpopulations. **(C)** Heatmap of differentially activated pathway between M1 and M2 macrophages. **(D-G)** Flow cytometric analysis and IHC staining of CD86 (M1 TAM-like marker) or CD206 (M2 TAM-like marker) expression in WT and ENO1-KO tumors (*n* = 6 per group). Two-side unpaired Student’s t-test. Data are presented as mean ± SD. **(H)** Flow cytometric analysis of CD206 expression in M1-polarized BMDMs cocultured with WT or ENO1-KO cells. *n* = 3 biologically independent samples per group. Two-side unpaired Student’s t-test. Data are presented as mean ± SD. **(I)** The mRNA expression of M2 TAM-like marker gene (CD206, Arg1) in M1-polarized BMDMs cocultured with WT or ENO1-KO cells. *n* = 3 biologically independent samples per group. Two-side unpaired Student’s t-test. Data are presented as mean ± SD. **(J)** Violin plot showing the SPP1 mRNA expression in CD11b^+^ macrophage from WT versus ENO1-KO tumors. **(K)** The mRNA expression of M2 TAM-like marker gene (CD206, Arg1) in M1-polarized BMDMs cocultured with or without mouse SPP1 recombinant protein. *n* = 3 biologically independent samples per group. Two-side unpaired Student’s t-test. Data are presented as mean ± SD. **(L)** The mRNA expression of M2 TAM-like marker gene (CD206, Arg1) and M1-like marker gene (CD86, IL-1α) in M1-polarized BMDMs cocultured with WT or ENO1-KO cells, with or without SPP1 overexpression. *n* = 3 biologically independent samples per group. One-way ANOVA with Dunnett’s multiple comparisons test. Data are presented as mean ± SD. **(M**,** N)** Flow cytometric analysis of CD206 expression in M1-polarized BMDMs cocultured with WT or ENO1-KO cells, with or without SPP1 overexpression or recombinant mouse SPP1 protein. *n* = 3 biologically independent samples per group. One-way ANOVA with Dunnett’s multiple comparisons test. Data are presented as mean ± SD. **(O)** Flow cytometric analysis of tumor-infiltrating CD206^+^ macrophages in ENO1-KO or ENO1-KO + oeSPP1 tumor tissues (*n* = 5 per group). Two-side unpaired Student’s t-test. Data are presented as mean ± SD. **(P)** A schematic representation of a transwell coculture assay involving ex vivo programmed M1-polarized BMDMs combined with Vector or His-SPP1 MB49 cells. **(Q)** Co-immunoprecipitation assays of M1-polarized BMDMs were performed using anti-His antibody. *n* = 3 biologically independent samples per group. **(R)** Immunofluorescence of M1-polarized BMDMs cells with a His antibody (green), a ITGA4 or ITGB1 antibody (red), and a DAPI antibody (blue). Scale bar: 25 μm. *n* = 3 biologically independent samples per group. **(S)** Western blotting assay detected ITGA4 and ITGB1 protein expression in M1-polarized BMDMs with or without recombinant mouse SPP1 protein treatment. **(T)** Western blotting assay detected ITGA4 and ITGB1 protein expression in M1-polarized BMDMs cocultured with WT or ENO1-KO cells, with or without recombinant mouse SPP1 protein. *n* = 3 biologically independent samples per group. **(U)** Flow cytometric analysis of CD206 expression in siITGA4 or siITGB1 M1-polarized BMDMs treated with recombinant mouse SPP1 protein. *n* = 3 biologically independent samples per group. **(V)** Flow cytometric analysis of CD206 expression in siITGA4 or siITGB1 M1-polarized BMDMs cocultured with MB49 cells. *n* = 3 biologically independent samples per group. Two-side unpaired Student’s t-test. Data are presented as mean ± SD. **p < 0.01, ***p < 0.001, ****p < 0.0001

Next, an additional potential parameter was investigated to distinguish between immunosuppressed WT and ENO1-KO tumors: TAMs and myeloid cells. These cells were identified based on the presence of the CD11b cell surface marker. In the TME, tumors often recruit or shape myeloid cells into TAMs, particularly of the M2-like immunosuppressive phenotype. We identified two subclusters of TAMs: antitumoral (M1) macrophages and protumoral (M2) macrophages (Fig. 5A). The proportion of antitumoral macrophages was higher in ENO1-KO tumors (Fig. 5B). KEGG enrichment analysis revealed upregulation of proinflammatory pathways (such as PD-L1 expression and PD-1 checkpoint pathway, Th17 cell differentiation, Th1 and Th2 cell differentiation, T cell receptor signaling pathway) in M1 macrophages. Conversely, anti-inflammatory pathways (such as IL-17 signaling pathway, Neutrophil extracellular trap formation, HIF-1 signaling pathway, JAK-STAT signaling pathway, and so on) were enriched in M2 macrophages (Fig. 5C). Analysis of single-cell RNA sequencing (scRNA-seq) data also revealed that markers of M1-like TAMs (CD86 and CD68) were highly expressed in ENO1-KO tumors. In contrast, markers of M2-like TAMs (CD206 and Arg1) were highly expressed in WT tumors (Additional file4A). Additionally, the TAM phenotype was assessed using flow cytometry and IHC staining of WT and ENO1-KO tumors for CD86 and CD206. The differential expression levels suggested higher numbers of M2-like TAMs in WT tumors, but M1-like TAMs were more prevalent in ENO1-KO tumors (Fig. 5D-G).

Subsequently, a transwell coculture assay was established to evaluate the impact of cancer cells on BMDMs or THP-1 cells programmed ex vivo to acquire an M1-like phenotype. The flow cytometry analysis of the M1-polarized BMDMs or THP-1 cells from the transwell assay revealed that WT cells exhibited a significant induction of CD206 expression compared to ENO1-KO or shENO1 cells (Fig. 5H, Additional file4B). Moreover, mRNA levels of CD206 and Arg1 were preferentially upregulated in M1-polarized BMDMs or THP-1 cells by WT or shNC cells (Fig. 5I, Additional file4C). Immunofluorescence staining for M1-like BMDMs using a CD206 antibody validated these results (Additional file4D). Considering the evident M2-like TAM programming activity observed in ENO1-WT cells, potential pathways contributing that contributed to this induction were investigated. Upon revisiting the scRNA-seq data, the CellChat analysis uncovered that BC cells secrete SPP1, which has the potential to interact with the integrin α4/β1 protein of macrophages to influence the polarization of TAMs in the TME (Fig. 4D). Furthermore, scRNA-seq data indicated a significant reduction in SPP1^+^ macrophages in ENO1-KO tumors compared to WT tumors (Fig. 5J). To investigate the effect of tumor-secreted SPP1 on TAM polarization, M1-polarized BMDMs or THP-1 cells were treated with recombinant mouse or human SPP1 protein, resulting in upregulation of CD206 and Arg1 expression (Fig. 5K, Additional file4E). Immunofluorescence staining demonstrated coexpression of SPP1 and CD206 in M1-polarized BMDMs (Additional file4F). In the transwell assay, SPP1 in ENO1-KO or shENO1 cells were overexpressed and cocultured M1-polarized BMDMs or THP-1 cells. Forced expression of SPP1 in ENO1-KO or shENO1 cells resulted in upregulation of CD206 and Arg1 expression and downregulation of CD86 and IL-1α expression (Fig. 5L, Additional file4G). Flow cytometry analysis and immunofluorescence staining confirmed that overexpression of SPP1 in ENO1-KO or shENO1 cells promoted CD206 expression in M1-polarized BMDMs (Fig. 5M, N, Additional file4H, I). Moreover, in vivo experiments demonstrated that upregulation of SPP1 in ENO1-KO cancer cells enhanced CD206^+^ TAMs infiltration in TME (Fig. 5O).

Additional analysis was conducted to determine whether the SPP1 protein produced by tumor can bind to the integrin α4/β1 protein in M1-polarized BMDMs or THP-1 cells. The results of co-IP assays demonstrated that the His-tagged SPP1 secreted by BC cells can interact with the ITGA4/ITGB1 in M1-polarized BMDMs or THP-1 cells (Fig. 5P, Q, Additional file4J, K). Immunofluorescence demonstrated the co-localization of His-tagged SPP1 and ITGA4/ITGB1 in M1-polarized BMDMs (Fig. 5R). Moreover, recombinant SPP1 protein could enhanced ITGA4/ITGB1 expression in M1-polarized BMDMs (Fig. 5S) and M1-polarized THP-1 cells (Additional file4L). In the coculture experiments, recombinant SPP1 protein could rescue ITGA4/B1 expression in M1-polarized macrophages that cocultured with ENO1-KO or shENO1 cells (Fig. 5T, Additional file4M). The flow cytometry analysis showed that knockdown of ITGA4 or ITGB1 can inhibit the effect of recombinant SPP1 protein or cancer cells on M2 TAM polarization (Fig. 5U, V, Additional file4N, O). These results revealed that tumor-intrinsic ENO1 triggers the transformation of M1-polarized macrophages into an M2-like immunosuppressive phenotype through the SPP1- ITGA4/B1 signaling pathway in the TME.

### M2 TAMs inhibits the function of CD8^+^ T cells via SPP1

**Fig. 6:**
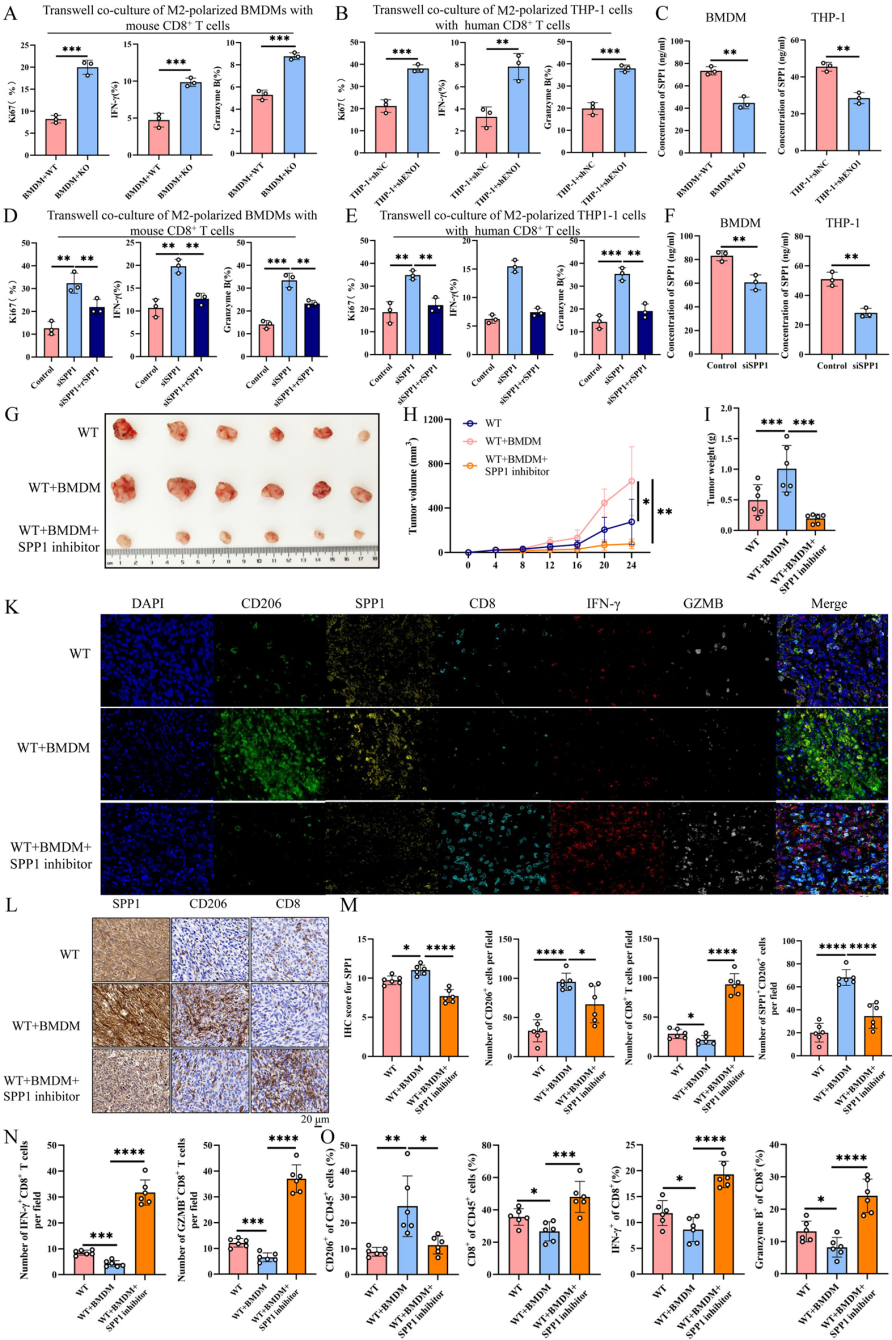
M2 TAMs inhibits the function of CD8^**+**^ T cells via SPP1. **A**,** B)** Flow cytometry analysis showed Ki67, IFN-γ, GZMB expression of mouse/human CD8 + T cells that were cocultured with M2-polarized BMDM (**A**) or M2-polarized THP-1 cells (B). *n* = 3 biologically independent samples per group. Two-side unpaired Student’s t-test. Data are presented as mean ± SD. **(C)** The ELISA assay detected the concentration of SPP1 in the coculture system. *n* = 3 biologically independent samples per group. Two-side unpaired Student’s t-test. Data are presented as mean ± SD. **(D**,** E)** Flow cytometry analysis showed Ki67, IFN-γ, GZMB expression of mouse/human CD8 + T cells that were cocultured with siSPP1 M2-polarized BMDM or M2-polarized THP-1 cells with or without recombinant spp1 protein. *n* = 3 biologically independent samples per group. One-way ANOVA with Dunnett’s multiple comparisons test. Data are presented as mean values ± SD. **(F)** The ELSA assay detected SPP1 concentration in the supernatant of M2-polarized BMDM (A) or M2-polarized THP-1 cells. *n* = 3 biologically independent samples per group. Two-side unpaired Student’s t-test. Data are presented as mean ± SD. **(G-I)** C57BL/6J mice (*n* = 6 per group) were subcutaneously co-injected with MB49 cells and M2-ploraized BMDM. Mice received intraperitoneally treated with 10 mg/Kg SPP1 inhibitor every other day after tumor inoculation. Tumor sizes (**G**), volumes (**H**), and weight (**I**) were measured. Two-way ANOVA with Tukey’s multiple comparison test. Data are presented as mean values ± SD. **(K-N)** Representative images of mIHC and IHC staining for SPP1, CD206, CD8, IFN-γ, GZMB in WT or ENO1-KO tumor tissues (*n* = 6 per group). Expression levels of the indicated proteins displayed. Two-way ANOVA with Tukey’s multiple comparison test. Data are presented as mean ± SD. **(O)** Flow cytometric analysis of tumor-infiltrating CD206^+^ cells, CD8^+^ T cells, IFN-γ^+^ or GZMB^+^ CD8^+^ T cells in distinct tumor tissues (*n* = 6 per group). Two-way ANOVA with Tukey’s multiple comparison test. Data are presented as mean ± SD. *p < 0.05, **p < 0.01, ***p < 0.001, ****p < 0.0001

Previous studies showed that M2 TAMs could secret SPP1 protein to promote the progression of cancers [16, 17]. We further investigate whether M2 TAMs could mediate the function of CD8^+^ T cells by secreting SPP1.To evaluate the impact of cancer cells-induced TAM polarization on the proliferation and activity of CD8^+^ T cells, coculture experiments was performed using BMDMs derived from either WT or ENO1-KO cancer cells along with CD8^+^ T cells. BMDMs or THP-1 cells from WT cancer cells suppressed the proportion of activated CD8^+^ T cells, as indicated by Ki67, IFN-γ, and GZMB expression, compared to those from ENO1-KO cancer cells (Fig. 6A, B). The ELISA assay showed that BMDMs or THP-1 cells from WT cancer cells could secrete higher SPP1 than those from ENO1-KO cancer cells (Fig. 6C). In addition, knockdown of SPP1 could suppress the inhibition effect of M2 TAMs on CD8^+^ T cells function, as indicated by Ki67, IFN-γ, and GZMB expression, while recombinant SPP1 protein can rescue it (Fig. 6D, E). The ELISA assay demonstrated that knockdown of SPP1 in M2 TAMs significantly inhibited the concentration of SPP1 protein in M2 TAMs supernatant (Fig. 6F). In vivo, BMDM and MB49 cell were co-injected into mice and treated with 10 mg/kg SPP1 inhibitor every other day. The results showed that SPP1 inhibitory can significantly inhibited the tumor growth and tumor weight (Fig. 6G-I). mIHC, IHC staining and flow cytometry analysis revealed that SPP1 inhibitory significantly decreased SPP1 expression and SPP1^+^CD206^+^ TAMs and increased the infiltration of CD8^+^ T cells, IFN-γ^+^ CD8^+^ T cells, and GZMB^+^ CD8^+^ T cells in the TME (Fig. 6K-O). These results demonstrated that M2 TAMs could inhibit the infiltration and activity of CD8^+^ T cells via SPP1 in the TME.

### ENO1 enhances the stability of SPP1 mRNA in BC cells

**Fig. 7:**
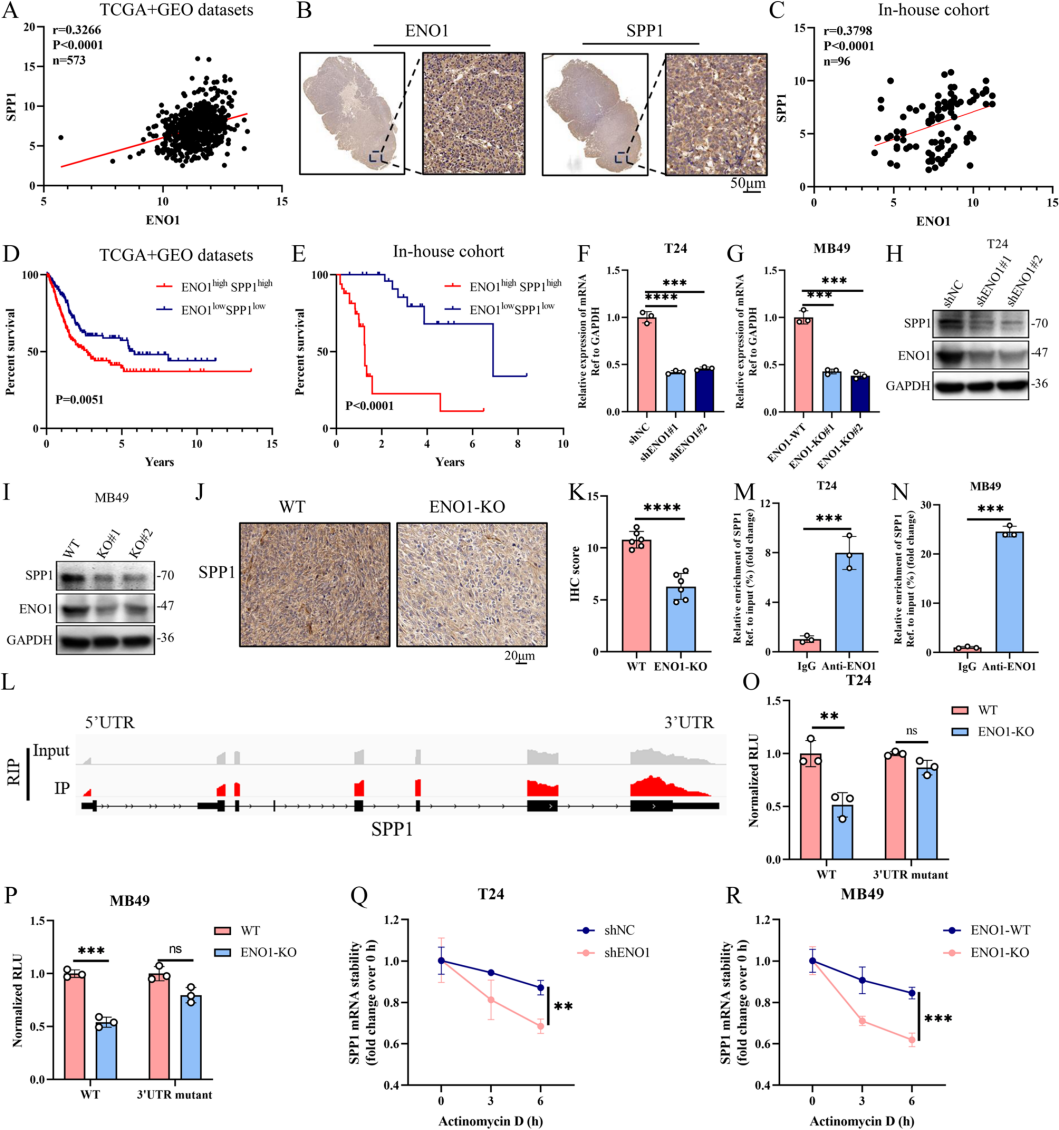
ENO1 enhances the stability of SPP1 mRNA in BC cells. **(A)** Correlation analysis between ENO1 expression and SPP1 expression in the TCGA and GEO datasets. Two-tailed Spearman correlation is reported. **(B)** Representative images of IHC staining for ENO1 and SPP1 in human BC samples. Scale bars: 50 μm. **(C)** Correlation between protein levels of ENO1 and SPP1 was determined by IHC staining. Two-tailed Spearman correlation is reported. **(D)** Kaplan-Meier curves of overall survival for BC patients stratified by ENO1 and SPP1 levels in the TCGA and GEO datasets. Data were analyzed using the log-rank test. **(E)** Kaplan-Meier curves of overall survival for BC patients stratified by ENO1 and SPP1 expression levels in our in-house cohort. Data were analyzed using the log-rank test. **(F-I)** Knockdown of ENO1 significant inhibit the SPP1 mRNA expression and protein expression in MB49 cells and T24 cells. *n* = 3 biologically independent samples per group. Two-side unpaired Student’s t-test. Data are presented as mean ± SD. **(J**,** K)** IHC staining of SPP1 expression in WT and ENO1-KO tumors (*n* = 6 per group). Two-side unpaired Student’s t-test. Data are presented as mean ± SD. **(L)** Distribution of ENO1-binding peaks across SPP1 by integrative genomics Viewer. **(M**,** N)** RIP analyses of MB49 cells or T24 cells were performed with an anti-ENO1 antibody followed by qPCR analyses with primer against SPP1 mRNA. *n* = 3 biologically independent samples per group. Two-side unpaired Student’s t-test. Data are presented as mean ± SD. **(O)** Luciferase with the WT or mutated 3’UTR site in the SPP1 gene was transfected into shNC or shENO1 T24 cells Relative luciferase activity was measured. *n* = 3 biologically independent samples per group. Two-side unpaired Student’s t-test. Data are presented as mean ± SD. **(P)** Luciferase with the WT or mutated 3’UTR site in the SPP1 gene was transfected into WT or ENO1-KO MB49 cells Relative luciferase activity was measured. *n* = 3 biologically independent samples per group. Two-side unpaired Student’s t-test. Data are presented as mean ± SD. **(Q**,** R)** Knockdown of ENO1 to detect the SPP1 mRNA expression levels in MB49 cells or T24 cells that treated with actinomycin D for the indicated time. *n* = 3 biologically independent samples per group. Two-side unpaired Student’s t-test. Data are presented as mean ± SD. **p < 0.01, ***p < 0.001, ****p < 0.0001

We next elucidated the molecular mechanism by which ENO1 regulates SPP1 expression in BC cells. A positive correlation between the ENO1 expression and SPP1 expression in the TCGA and GEO BC cohorts (Fig. 7A). We further examined the expression level of ENO1 and SPP1 in human BC tissues by using IHC. The result also demonstrated that ENO1 protein level was positively correlated with SPP1 protein level in BC tissues (Fig. 7B, C). Importantly, combined high level of ENO1 and SPP1 or ENO1 and PD-L1 was more strongly related to the poor prognosis of BC patients (Fig. 7D, E). Moreover, inhibition of ENO1 significantly suppressed SPP1 mRNA and protein expression in MB49 and T24 cells (Fig. 7F-I). In vivo experiments also showed the SPP1 expression was significantly downregulated in ENO1-KO tumor tissues (Fig. 7J, K). Given that ENO1 can act as an RNA-binding protein to regulate RNA degradation, an additional experiment was conducted to examine its involvement in the degradation of SPP1 mRNA, which affects SPP1 expression. RIP-seq found the peaks fit well with the ENO1-binding site at the 3′ untranslated region (3′UTR) of SPP1, as shown by the Integrative Genomics Viewer (Fig. 7L). RIP assay confirmed the binding of ENO1 protein to SPP1 mRNA in MB49 and T24 cells (Fig. 7M, N). Dual-luciferase assays revealed that the activity of ENO1 mRNA 3′UTR site was significantly inhibited in the ENO1-KO cells or shENO1 cells (Fig. 7O, P). Furthermore, SPP1 mRNA was significantly degraded in ENO1-KO cells in the presence of actinomycin D (Fig. 7Q, R), indicating that ENO1 positively regulated the SPP1 expression by enhancing its mRNA stability.

### ENO1 is a therapeutic target for cancer immunotherapy

**Fig. 8:**
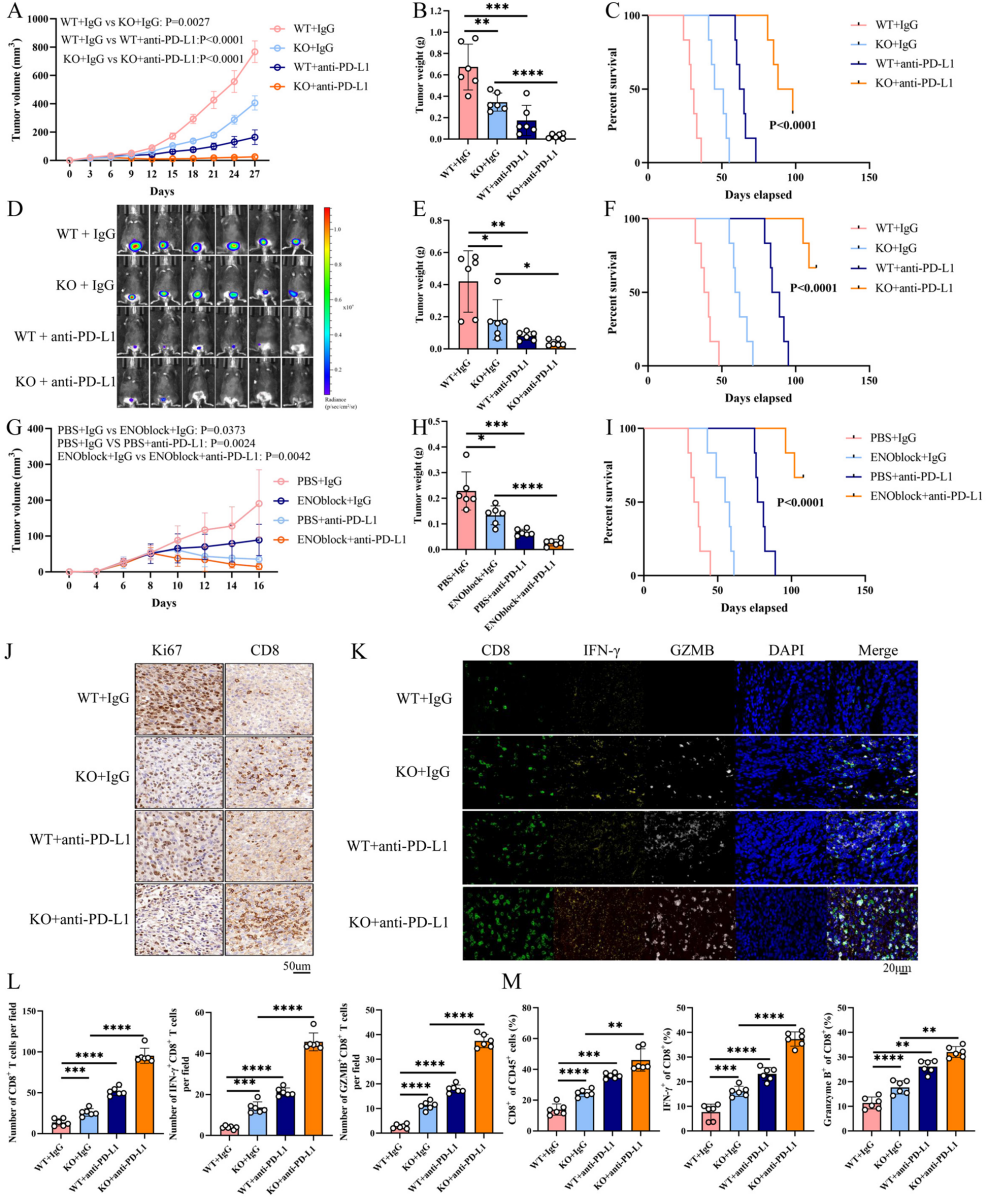
ENO1 is a therapeutic target for cancer immunotherapy. **(A-C)** C57BL/6J mice (*n* = 6 per group) were subcutaneously inoculated with WT or ENO1-KO cells. Mice received intraperitoneally treated with 200 µg anti-PD-L1 or IgG on days 7, 9, 11, 13, and 15 after tumor inoculation. Tumor volumes (**A**), weight (**B**), and mice survival time (**C**) were measured. Two-way ANOVA with Tukey’s multiple comparison test. Data are presented as mean values ± SD. Tumor volumes exceeding 1500mm3 were considered events. Two-sided log-rank test. **(D-F)** In vivo bioluminescence imaging of bladder orthotopic tumor model. Luciferase-labeled WT or ENO1 MB49 cells were subcutaneously inoculated into the bladder of C57BL/6J mice (*n* = 6 per group). Mice were intraperitoneally treated with 200 µg anti-PD-L1 or IgG on days 7, 9, 11, and 13 after tumor inoculation. The signals were measured on 18 days using an IVIS Spectrum In Vivo imaging system (**D**), and tumor volumes (**E**) and mice survival time were measured. Two-way ANOVA with Tukey’s multiple comparison test. Data are presented as mean ± SD. **(G-I)** MB49 cells were subcutaneously inoculated into C57BL/6J mice and then treatment with ENOblock and anti-PD-L1 (*n* = 6 per group). Tumor volumes (**G**), weight (**H**) and mice survival time (**I**) were measured. Two-way ANOVA with Tukey’s multiple comparison test. Data are presented as mean ± SD. Tumor volumes exceeding 1500mm3 were considered events. Two-sided log-rank test. **(J-L)** Representative images of IHC and mIHC staining for Ki67, CD8, IFN-γ, GZMB in different tumor tissues (*n* = 6 per group). Expression levels of the indicated proteins displayed. Two-way ANOVA with Tukey’s multiple comparison test. Data are presented as mean ± SD. **(M)** Flow cytometric analysis of tumor-infiltrating CD4^+^ T cells, CD8^+^ T cells, IFN-γ^+^ or GZMB^+^ CD8^+^ T cells in distinct tumor tissues (*n* = 6 per group). Two-way ANOVA with Tukey’s multiple comparison test. Data are presented as mean ± SD. *p < 0.05, **p < 0.01, ***p < 0.001, ****p < 0.0001

Considering the enhanced infiltration and function of T cells in the TME upon ENO1 depletion, whether targeting ENO1 could enhance anti-PD-L1 therapy in BC was further investigated. Mice injected with WT and ENO1-KO cells were treated with anti-PD-L1, revealing that ENO1 knockdown significantly augmented the efficacy of anti-PD-L1 treatment and prolong the survive time of mice (Fig. 8A-F, Additional file5A, B). Moreover, ENOblock significantly inhibited tumor growth and prolong the survive time of mice when combined with anti-PD-L1 treatment (Fig. 8G-I, Additional file5C). IHC staining, mIHC, and flow cytometry analysis revealed that this combined therapy significantly increased the infiltration of CD8^+^ T cells, IFN-γ^+^ CD8^+^ T cells, and GZMB^+^ CD8^+^ T cells, within the tumors (Fig. 8J-M).

## Discussion

Evading immune destruction has emerged as a hallmark of cancer, posing a significant challenge to the development of effective anticancer therapies [18, 19]. In our study, in vivo genome-wide CRISPR screening and RNA-seq for clinical BC tissues identified ENO1 as a regulator of antitumor-tumor immunity and sensitivity to anti-PD-L1 therapy. Further studies indicated a strong association between ENO1 overexpression and immunosuppression in BC, as evidenced by reduced infiltration of CD8^+^ T cells and resistance to anti-PD-L1 therapy. The in vitro and in vivo experiments demonstrated that tumor-intrinsic ENO1 deficiency impedes tumorigenesis and reshapes the immune landscape of the TME by facilitating the infiltration of CD8^+^ T cells, suggesting a pivotal role for ENO1 in regulating the tumor immune microenvironment (TIME).

The TIME can be divided into two categories: infiltrated–excluded also known as “Cold tumors”, where CD8^+^T cells are prevented from entering the tumor core, and infiltrated–inflamed also known as “Hot tumors”, which are characterized by highly activated CD8^+^ T cells that express IFN-γ [20–22]. A single-cell RNA sequencing analysis was used to identify distinct patterns of immune cell infiltration in tumors from WT versus ENO1-KO tumors. Remarkably, ENO1-KO tumors exhibited a heightened abundance of CD8^+^ T cells, mainly proliferating and IFN-responsive subsets, indicative of augmented antitumor immunity. Conversely, WT tumors favored the recruitment of immunosuppressive myeloid cells, particularly M2 TAMs, which known to dampen T-cell-mediated immune responses and enhance cancer cell resistance against immunotherapy [23, 24]. Moreover, the current investigation identified SPP1 as a downstream effector of ENO1, mediating its immunosuppressive effects on CD8^+^T cells. By upregulating SPP1 expression, ENO1 promoted the polarization of M1-like TAMs towards an M2-like phenotype. ENO1 deficiency significantly reduced the SPP1^+^ macrophage subtype in TME, indicating that inhibition of immunoinhibitory gene expression in macrophages may contribute to the recruitment of CD8^+^ T cells to establish an immune-inflamed TME. Previous studies have also shown that SPP1 expression in the TME regulated TAM polarity and significantly correlated with immune cell profiles, antitumor factors, and patient outcomes [17, 25]. Moreover, our study found that M2 TAM also could secrete SPP1 to inhibit the function of CD8^+^ T cells. Mechanistically, SPP1 can interact with ITGA4/B1 to promote the M2 TAM polarization and inhibit CD8^+^T cell function in the TME. Zheng et al. found that SPP1 can directly interact with ITGB1 to orchestrate Th17 cell differentiation via the ERK signaling pathway in metabolic dysfunction-associated steatotic liver disease [26]. These results indicated that the crosstalk among cancer cells, CD8^+^T cells, and TAMs remodeling the tumor immune microenvironment via the SPP1- ITGA4/ITGB1 pathway in TME.

Importantly, therapeutic interventions targeting oncogenes demonstrate promising outcomes in preclinical models, both as monotherapy and in conjunction with anti-PD-L1 therapy [27–29]. In the study, silencing ENO1 augments the efficacy of anti-PD-L1 treatment, resulting in heightened infiltration and activation of CD8^+^ T cells within the TME. These results underscore the therapeutic potential of targeting ENO1 to counter immune evasion and enhance the efficacy of immunotherapy in BC. However, the primary limitation of this study is that it focuses mainly on the impact of ENO1 on the function and polarization of CD8^+^ T cells and macrophages within the tumor microenvironment, without exploring the broader molecular mechanisms through which ENO1 regulates these immune cells. Moreover, the study does not investigate whether ENO1 interacts with other immune cells to reshape the tumor immune microenvironment, potentially influencing the effectiveness of immunotherapy. Future research will therefore aim to further investigate the molecular mechanisms by which ENO1 contributes to tumor immune evasion.

## Conclusions

In conclusion, the current study elucidated the intricate mechanisms underlying ENO1-mediated immune evasion in BC and suggested targeting ENO1 as a novel therapeutic strategy to enhance antitumor immunity and improve clinical outcomes in BC patients. Further elucidation of the precise molecular pathways regulated by ENO1 and its interactions with the TME is warranted to fully exploit its therapeutic potential.

## Electronic supplementary material

Below is the link to the electronic supplementary material.

Supplementary Material 1

## Data Availability

No datasets were generated or analysed during the current study.

## References

[1] Bray F, Laversanne M, Sung H, Ferlay J, Siegel RL, Soerjomataram I, Jemal A. Global cancer statistics 2022: GLOBOCAN estimates of incidence and mortality worldwide for 36 cancers in 185 countries. CA Cancer J Clin. 2024 Apr;4. 10.3322/caac.21834. 10.3322/caac.21834 38572751

[2] Kamat AM, Apolo AB, Babjuk M, Bivalacqua TJ, Black PC, Buckley R, Campbell MT, Compérat E, Efstathiou JA, Grivas P, Gupta S, Kurtz NJ, Lamm D, Lerner SP, Li R, McConkey DJ, Palou Redorta J, Powles T, Psutka SP, Shore N, Steinberg GD, Sylvester R, Witjes JA, Galsky MD. Definitions, end points, and clinical trial designs for bladder cancer: recommendations from the society for immunotherapy of Cancer and the international bladder Cancer group. J Clin Oncol. 2023;41(35):5437–47. 10.1200/JCO.23.00307. 37793077 10.1200/JCO.23.00307 PMC10713193

[3] Szeto GL, Finley SD. Integrative approaches to Cancer immunotherapy. Trends Cancer. 2019;5(7):400–10. 10.1016/j.trecan.2019.05.010. 31311655 10.1016/j.trecan.2019.05.010 PMC7467854

[4] Zhang Y, Zhang Z. The history and advances in cancer immunotherapy: Understanding the characteristics of tumor-infiltrating immune cells and their therapeutic implications. Cell Mol Immunol. 2020;17(8):807–21. 10.1038/s41423-020-0488-6. 32612154 10.1038/s41423-020-0488-6 PMC7395159

[5] Oliveira G, Wu CJ. Dynamics and specificities of T cells in cancer immunotherapy. Nat Rev Cancer. 2023;23(5):295–316. 10.1038/s41568-023-00560-y. 37046001 10.1038/s41568-023-00560-y PMC10773171

[6] Chow A, Perica K, Klebanoff CA, Wolchok JD. Clinical implications of T cell exhaustion for cancer immunotherapy. Nat Rev Clin Oncol. 2022;19(12):775–90. 10.1038/s41571-022-00689-z. 36216928 10.1038/s41571-022-00689-z PMC10984554

[7] Tichet M, Wullschleger S, Chryplewicz A, Fournier N, Marcone R, Kauzlaric A, Homicsko K, Deak LC, Umaña P, Klein C, Hanahan D. Bispecific PD1-IL2v and anti-PD-L1 break tumor immunity resistance by enhancing stem-like tumor-reactive CD8 + T cells and reprogramming macrophages. Immunity. 2023;56(1):162–e1796. 10.1016/j.immuni. 36630914 10.1016/j.immuni.2022.12.006

[8] Sun L, Suo C, Zhang T, Shen S, Gu X, Qiu S, Zhang P, Wei H, Ma W, Yan R, Chen R, Jia W, Cao J, Zhang H, Gao P. ENO1 promotes liver carcinogenesis through YAP1-dependent arachidonic acid metabolism. Nat Chem Biol. 2023;19(12):1492–503. 10.1038/s41589-023-01391-6. 37500770 10.1038/s41589-023-01391-6

[9] Zhang T, Sun L, Hao Y, Suo C, Shen S, Wei H, Ma W, Zhang P, Wang T, Gu X, Li ST, Chen Z, Yan R, Zhang Y, Cai Y, Zhou R, Jia W, Huang F, Gao P, Zhang H. ENO1 suppresses cancer cell ferroptosis by degrading the mRNA of iron regulatory protein 1. Nat Cancer. 2022;3(1):75–89. 10.1038/s43018-021-00299-1. 35121990 10.1038/s43018-021-00299-1

[10] Li HJ, Ke FY, Lin CC, Lu MY, Kuo YH, Wang YP, Liang KH, Lin SC, Chang YH, Chen HY, Yang PC, Wu HC. ENO1 promotes lung Cancer metastasis via HGFR and WNT Signaling-Driven Epithelial-to-Mesenchymal transition. Cancer Res. 2021;81(15):4094–109. 10.1158/0008-5472.CAN-20-3543. 34145039 10.1158/0008-5472.CAN-20-3543

[11] Zhu Q, Li J, Sun H, et al. O-GlcNAcylation of enolase 1 serves as a dual regulator of aerobic Glycolysis and immune evasion in colorectal cancer. Proc Natl Acad Sci U S A. 2024;121(44):e2408354121. 39446384 10.1073/pnas.2408354121 PMC11536113

[12] Shen C, Liu J, Xie F, Yu Y, Ma X, Hu D, Liu C, Wang Y. N6-Methyladenosine enhances the translation of ENO1 to promote the progression of bladder cancer by inhibiting PCNA ubiquitination. Cancer Lett. 2024;595:217002. 10.1016/j.canlet.2024.217002. 38823761 10.1016/j.canlet.2024.217002

[13] Shen C, Liu J, Jiao W, Zhang X, Zhao X, Yang X, Wang Y. A feed-forward loop based on aerobic Glycolysis and TGF-β between tumor-associated macrophages and bladder cancer cells promoted malignant progression and immune escape. J Cancer Res Clin Oncol. 2023;149(14):12867–80. 10.1007/s00432-023-05164-5. 37462772 10.1007/s00432-023-05164-5 PMC11798317

[14] Zhang X, Liu J, Yang X, Jiao W, Shen C, Zhao X, Wang Y. High expression of COL6A1 predicts poor prognosis and response to immunotherapy in bladder cancer. Cell Cycle. 2023;22(5):610–8. 10.1080/15384101.2022.2154551. 36474424 10.1080/15384101.2022.2154551 PMC9928451

[15] Chen Z, Zhou L, Liu L, Hou Y, Xiong M, Yang Y, Hu J, Chen K. Single-cell RNA sequencing highlights the role of inflammatory cancer-associated fibroblasts in bladder urothelial carcinoma. Nat Commun. 2020;11(1):5077. 10.1038/s41467-020-18916-5. 33033240 10.1038/s41467-020-18916-5 PMC7545162

[16] Bill R, Wirapati P, Messemaker M, Roh W, Zitti B, Duval F, Kiss M, Park JC, Saal TM, Hoelzl J, Tarussio D, Benedetti F, Tissot S, Kandalaft L, Varrone M, Ciriello G, McKee TA, Monnier Y, Mermod M, Blaum EM, Gushterova I, Gonye ALK, Hacohen N, Getz G, Mempel TR, Klein AM, Weissleder R, Faquin WC, Sadow PM, Lin D, Pai SI, Sade-Feldman M, Pittet MJ. CXCL9:SPP1 macrophage Polarity identifies a network of cellular programs that control human cancers. Science. 2023;381(6657):515–24. 10.1126/science.ade2292. 37535729 10.1126/science.ade2292 PMC10755760

[17] Su X, Liang C, Chen R, Duan S. Deciphering tumor microenvironment: CXCL9 and SPP1 as crucial determinants of tumor-associated macrophage Polarity and prognostic indicators. Mol Cancer. 2024;23(1):13. 10.1186/s12943-023-01931-7. 38217023 10.1186/s12943-023-01931-7 PMC10790255

[18] Ghorani E, Swanton C, Quezada SA. Cancer cell-intrinsic mechanisms driving acquired immune tolerance. Immunity. 2023;56(10):2270–95. 10.1016/j.immuni.2023.09.004. 37820584 10.1016/j.immuni.2023.09.004

[19] Garner H, de Visser KE. Immune crosstalk in cancer progression and metastatic spread: a complex conversation. Nat Rev Immunol. 2020;20(8):483–97. 10.1038/s41577-019-0271-z. 32024984 10.1038/s41577-019-0271-z

[20] Binnewies M, Roberts EW, Kersten K, Chan V, Fearon DF, Merad M, Coussens LM, Gabrilovich DI, Ostrand-Rosenberg S, Hedrick CC, Vonderheide RH, Pittet MJ, Jain RK, Zou W, Howcroft TK, Woodhouse EC, Weinberg RA, Krummel MF. Understanding the tumor immune microenvironment (TIME) for effective therapy. Nat Med. 2018;24(5):541–50. 10.1038/s41591-018-0014-x. 29686425 10.1038/s41591-018-0014-x PMC5998822

[21] Park J, Hsueh PC, Li Z, Ho PC. Microenvironment-driven metabolic adaptations guiding CD8 + T cell anti-tumor immunity. Immunity. 2023;56(1):32–42. 10.1016/j.immuni.2022.12.008. 36630916 10.1016/j.immuni.2022.12.008

[22] Zhang J, Huang D, Saw PE, Song E. Turning cold tumors hot: from molecular mechanisms to clinical applications. Trends Immunol. 2022;43(7):523–45. 10.1016/j.it.2022.04.010. 35624021 10.1016/j.it.2022.04.010

[23] Mantovani A, Allavena P, Marchesi F, Garlanda C. Macrophages as tools and targets in cancer therapy. Nat Rev Drug Discov. 2022;21(11):799–820. 10.1038/s41573-022-00520-5. 35974096 10.1038/s41573-022-00520-5 PMC9380983

[24] DeNardo DG, Ruffell B. Macrophages as regulators of tumour immunity and immunotherapy. Nat Rev Immunol. 2019;19(6):369–82. 10.1038/s41577-019-0127-6. 30718830 10.1038/s41577-019-0127-6 PMC7339861

[25] Qi J, Sun H, Zhang Y, Wang Z, Xun Z, Li Z, Ding X, Bao R, Hong L, Jia W, Fang F, Liu H, Chen L, Zhong J, Zou D, Liu L, Han L, Ginhoux F, Liu Y, Ye Y, Su B. Single-cell and Spatial analysis reveal interaction of FAP + fibroblasts and SPP1 + macrophages in colorectal cancer. Nat Commun. 2022;13(1):1742. 10.1038/s41467-022-29366-6. 35365629 10.1038/s41467-022-29366-6 PMC8976074

[26] Zheng Y, Zhao L, Xiong Z, Huang C, Yong Q, Fang D, Fu Y, Gu S, Chen C, Li J, Zhu Y, Liu J, Liu F, Li Y. Ursolic acid targets secreted phosphoprotein 1 to regulate Th17 cells against metabolic dysfunction-associated steatotic liver disease. Clin Mol Hepatol. 2024;30(3):449–67. 38623614 10.3350/cmh.2024.0047 PMC11261229

[27] Song H, Chen L, Pan X, Shen Y, Ye M, Wang G, Cui C, Zhou Q, Tseng Y, Gong Z, Zhong B, Cui H, Mo S, Zheng J, Jin B, Zheng W, Luo F, Liu J. Targeting tumor monocyte-intrinsic PD-L1 by rewiring STING signaling and enhancing STING agonist therapy. Cancer Cell. 2025;43(3):503–e51810. 40068600 10.1016/j.ccell.2025.02.014

[28] Morra F, Merolla F, Criscuolo D, Insabato L, Giannella R, Ilardi G, Cerrato A, Visconti R, Staibano S, Celetti A. CCDC6 and USP7 expression levels suggest novel treatment options in high-grade urothelial bladder cancer. J Exp Clin Cancer Res. 2019;38(1):90. 30786932 10.1186/s13046-019-1087-1 PMC6381716

[29] Criscuolo D, Morra F, Giannella R, Visconti R, Cerrato A, Celetti A. New combinatorial strategies to improve the PARP inhibitors efficacy in the urothelial bladder Cancer treatment. J Exp Clin Cancer Res. 2019;38(1):91. 30791940 10.1186/s13046-019-1089-z PMC6385418

