# Supplementary material for: Tumor-intrinsic ENO1 inhibition promotes antitumor immune response and facilitates the efficacy of anti-PD-L1 immunotherapy in bladder cancer
Source: J Exp Clin Cancer Res. 2025 Jul 15;44:207. doi: 10.1186/s13046-025-03464-x (PMC12261641; doi:10.1186/s13046-025-03464-x)
Supplement: Supplementary file 1 — Supplementary Material 1 [file 13046_2025_3464_MOESM1_ESM.pdf]

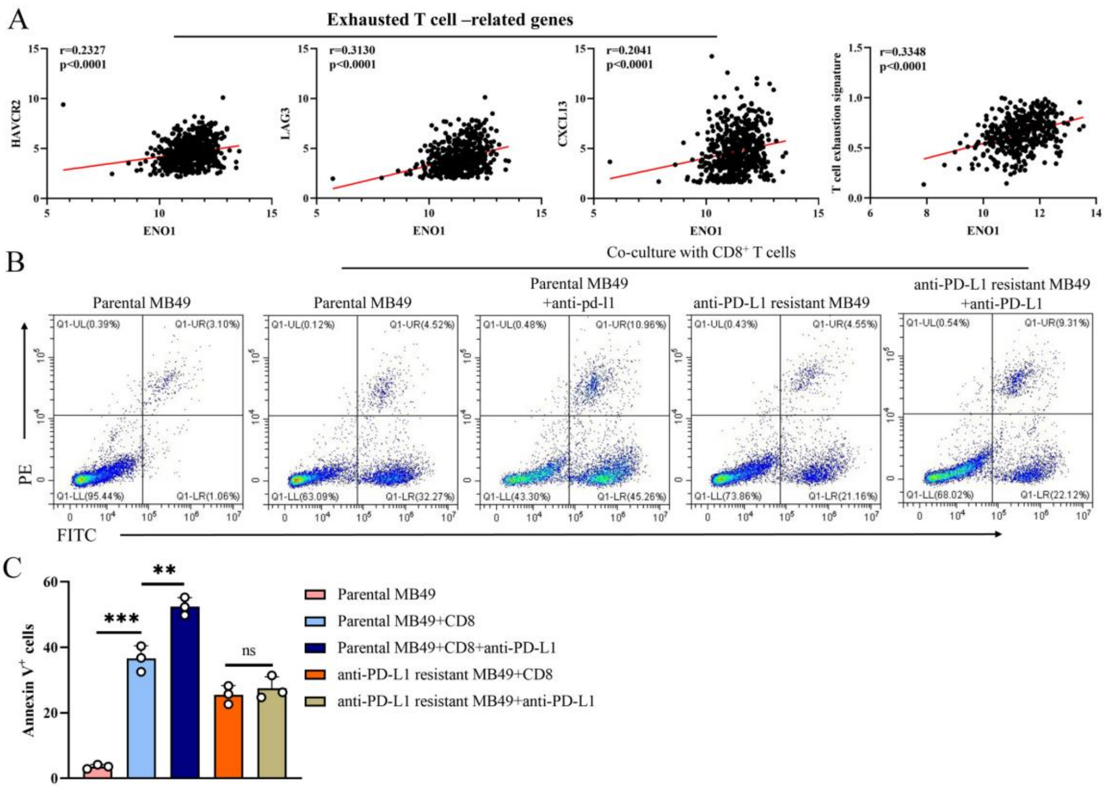

Additional file1. (A) Correlation analysis between ENO1 expression with exhausted T cells related markers (HAVCR2, LAG3, CXCL3) and T cell exhaustion signature in the TCGA bladder urothelial carcinoma (TCGA-BLCA) dataset. Two-tailed Spearman correlation is reported. **(B, C)** Flow cytometric analysis of $CD8^+$ T cell-mediated killing of parental MB49 cells and anti-PD-L1-resistant MB49 cells in the presence or absence of anti-PD-L1 antibody. n = 3 biologically independent samples per group. One-way ANOVA with Dunnett's multiple comparisons test. Data are presented as mean values ± SD.

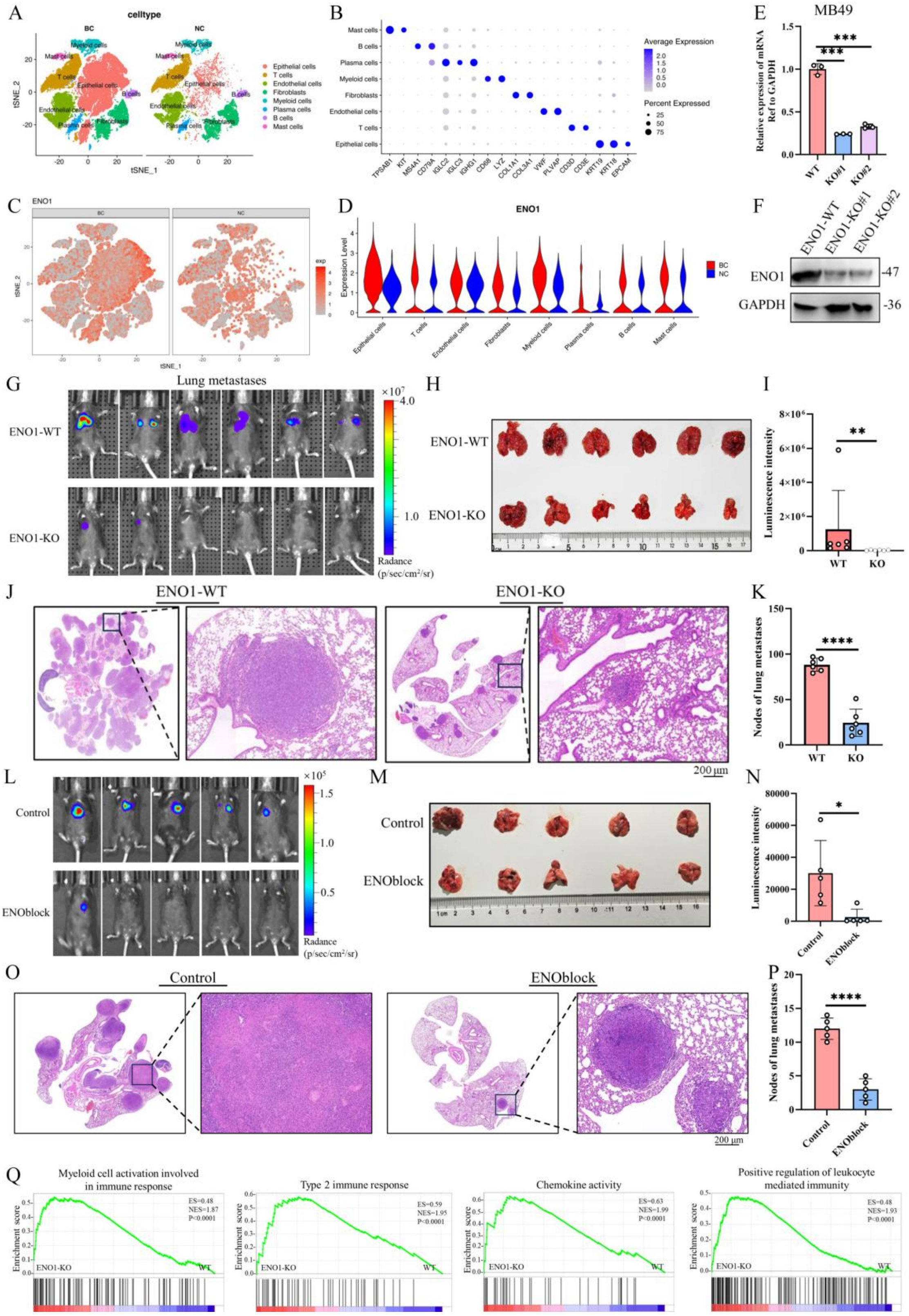

Additional file2. (A) The t-SNE plot displays all cell subpopulations analyzed from a public BC single-cell RNA sequencing dataset, color coded by cell cluster and cell type. **(B)** The expression levels of marker genes in each cell subpopulation are depicted. **(C, D)** The expression of ENO1 in each cell subpopulation between normal and BC tumor

samples. **(E-F)** RT-qPCR and western blotting analyses the expression level of ENO1 in WT or ENO1-KO cancer cells. n = 3 biologically independent samples per group. Two-side unpaired Student's t-test. Data are presented as mean ± SD. **(G-K)** In vivo bioluminescence imaging of lung metastases. Intravenous injection of luciferase-labeled WT or ENO1-KO MB49 cells into C57BL/6J mice via the tail vein (n=6 per group), followed by measurement of experimental lung metastases growth on day 20 using an IVIS Spectrum In Vivo imaging system (G, I). Images and hematoxylin & eosin staining of lung metastases (H, J) and statistical analysis of lung metastatic nodules (K). Scale bars: 200μm. Two-side unpaired Student's t-test. Data are presented as mean ± SD. **(L-P)** In vivo bioluminescence imaging of lung metastases. Intravenous injection of luciferase-labeled WT or ENO1-KO MB49 cells into C57BL/6J mice via the tail vein (n=5 per group), followed by treatment with 8mg/kg ENOblock and measurement of experimental lung metastases growth on day 14 using an IVIS Spectrum In Vivo imaging system (L, N). Images and hematoxylin & eosin staining of lung metastases (M, O) and statistical analysis of lung metastatic nodules (P). Scale bars: 200μm. Two-side unpaired Student's t-test. Data are presented as mean ± SD. **(Q)** GSEA revealing immune-associated pathways correlated with ENO1 expression in the RNA-seq dataset. $*p < 0.05$, $**p < 0.01$, $***p < 0.001$.

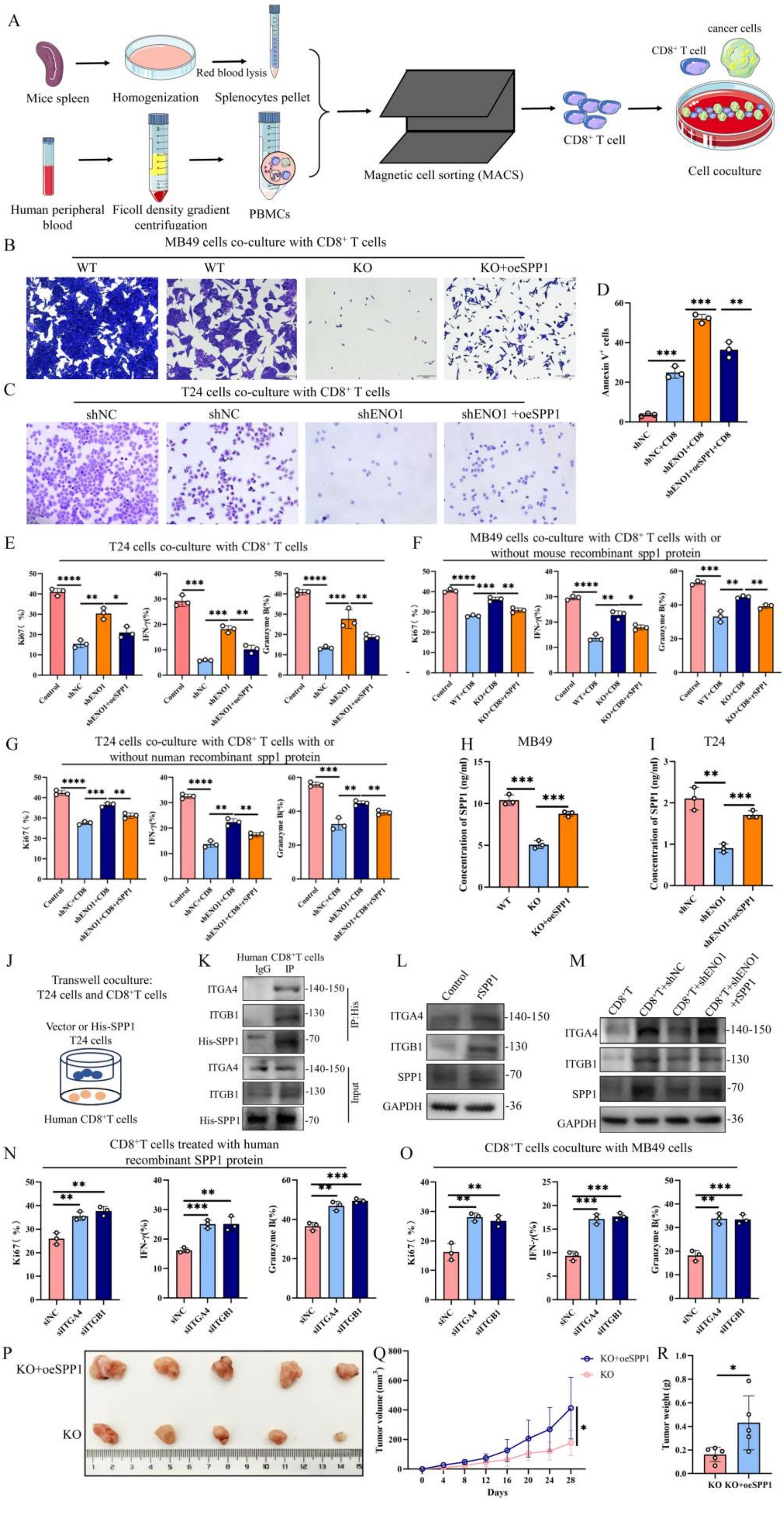
A
Mice spleen
Homogenization
Red blood lysis
Splenocytes pellet
Human peripheral blood
Ficoll density gradient centrifugation
PBMCs
Magnetic cell sorting (MACS)
CD8+ T cell
cancer cells
Cell coculture
B
MB49 cells co-culture with CD8+ T cells
WT
WT
KO
KO+oeSPP1
C
T24 cells co-culture with CD8+ T cells
shNC
shNC
shENO1
shENO1 +oeSPP1
D
Annexin V+ cells
shNC
shNC+CD8
shENO1+CD8
shENO1+oeSPP1+CD8
E
T24 cells co-culture with CD8+ T cells
Ki67 (%)
IFN-γ(%)
Granzyme B(%)
Control
shNC
shENO1
shENO1+oeSPP1
F
MB49 cells co-culture with CD8+ T cells with or without mouse recombinant spp1 protein
WT+CD8
KO+CD8
KO+CD8+rSPP1
G
T24 cells co-culture with CD8+ T cells with or without human recombinant spp1 protein
shNC+CD8
shENO1+CD8
shENO1+CD8+rSPP1
H
MB49
Concentration of SPP1 (ng/ml)
WT
KO
KO+oeSPP1
I
T24
shNC
shENO1
shENO1+oeSPP1
J
Transwell coculture: T24 cells and CD8+T cells
Vector or His-SPP1 T24 cells
Human CD8+T cells
K
Human CD8+T cells
IgG
IP
ITGA4
ITGB1
His-SPP1
-140-150
-130
-70
IP:His
Input
L
Control
rSPP1
ITGA4
ITGB1
SPP1
GAPDH
-36
M
CD8+T
CD8+T+shNC
CD8+T+shENO1
CD8+T+shENO1 +rSPP1
N
CD8+T cells treated with human recombinant SPP1 protein
siNC
siITGA4
siITGB1
O
CD8+T cells coculture with MB49 cells
P
KO+oeSPP1
KO
Q
Tumor volume (mm3)
Days
KO+oeSPP1
KO
R
Tumor weight (g)
KO
KO+oeSPP1

Additional file3. (A) Illustration shows the in vitro procedure of human and mouse $CD8^+$ T cells isolation and cocultivation with cancer cells. **(B)** Proliferation assay was conducted to evaluate WT, ENO1-KO, or ENO1-KO+oeSPP1 cells in the presence of mouse $CD8^+$ T cells. n = 3 biologically independent samples per group. **(C, D)** Proliferation and apoptosis assays were conducted to evaluate shNC, shENO1, or shENO1+oeSPP1 cells in the presence of human $CD8^+$ T cells. n = 3 biologically independent samples per group. One-way ANOVA with Dunnett's multiple comparisons test. Data are presented as mean values ± SD. **(E)** Flow cytometry analysis showed Ki67, IFN-γ, GZMB expression of mouse $CD8^+$ T cells that were cocultured with shNC, shENO1, shENO1+oeSPP1 cells. n = 3 biologically independent samples per group. One-way ANOVA with Dunnett's multiple comparisons test. Data are presented as mean values ± SD. **(F, G)** Flow cytometry analysis showed Ki67, IFN-γ, GZMB expression of mouse/human $CD8^+$ T cells that were cocultured with MB19 or T24 cells treated with mouse/human recombinant SPP1 protein. n = 3 biologically independent samples per group. One-way ANOVA with Dunnett's multiple comparisons test. Data are presented as mean values ± SD. **(H, I)** The ELISA assay detected the concentration of SPP1 in the coculture system. n = 3 biologically independent samples per group. One-way ANOVA with Dunnett's multiple comparisons test. Data are presented as mean values ± SD. **(J)** A schematic representation of a transwell coculture assay involving human $CD8^+$ T cells combined with His-SPP1 T24 cells. **(K)** Co-immunoprecipitation assays of $CD8^+$ T cells were performed using anti-His antibody. n = 3 biologically independent samples per group. **(L)** Western blotting assay detected ITGA4 and ITGB1 protein expression in human $CD8^+$ T cells with or without recombinant mouse SPP1 protein treatment. n = 3 biologically independent samples per group. **(M)** Western blotting assay detected ITGA4 and ITGB1 protein expression in human $CD8^+$ T cells cocultured with WT or ENO1-KO cells, with or without recombinant mouse SPP1 protein. n = 3 biologically independent samples per group. **(N)** Flow cytometric analysis of Ki67, IFN-γ and GZMB expression in siITGA4 or siITGB1 $CD8^+$ T cells treated with recombinant human SPP1 protein. n = 3 biologically independent samples per group. **(O)** Flow

cytometric analysis of Ki67, IFN-γ and GZMB expression in siITGA4 or siITGB1 $CD8^+$ T cells cocultured with T24 cells. n = 3 biologically independent samples per group. Two-side unpaired Student's t-test. Data are presented as mean ± SD. **(P-R)** ENO1-KO or ENO1-KO+oeSPP1 cells were subcutaneously inoculated into C57BL/6J mice (n=5 per group). Tumor sizes (O), volumes (P), and weight (Q) were measured. Two-side unpaired Student's t-test. Data are presented as mean ± SD. *p < 0.05, **p < 0.01, ***p < 0.001.

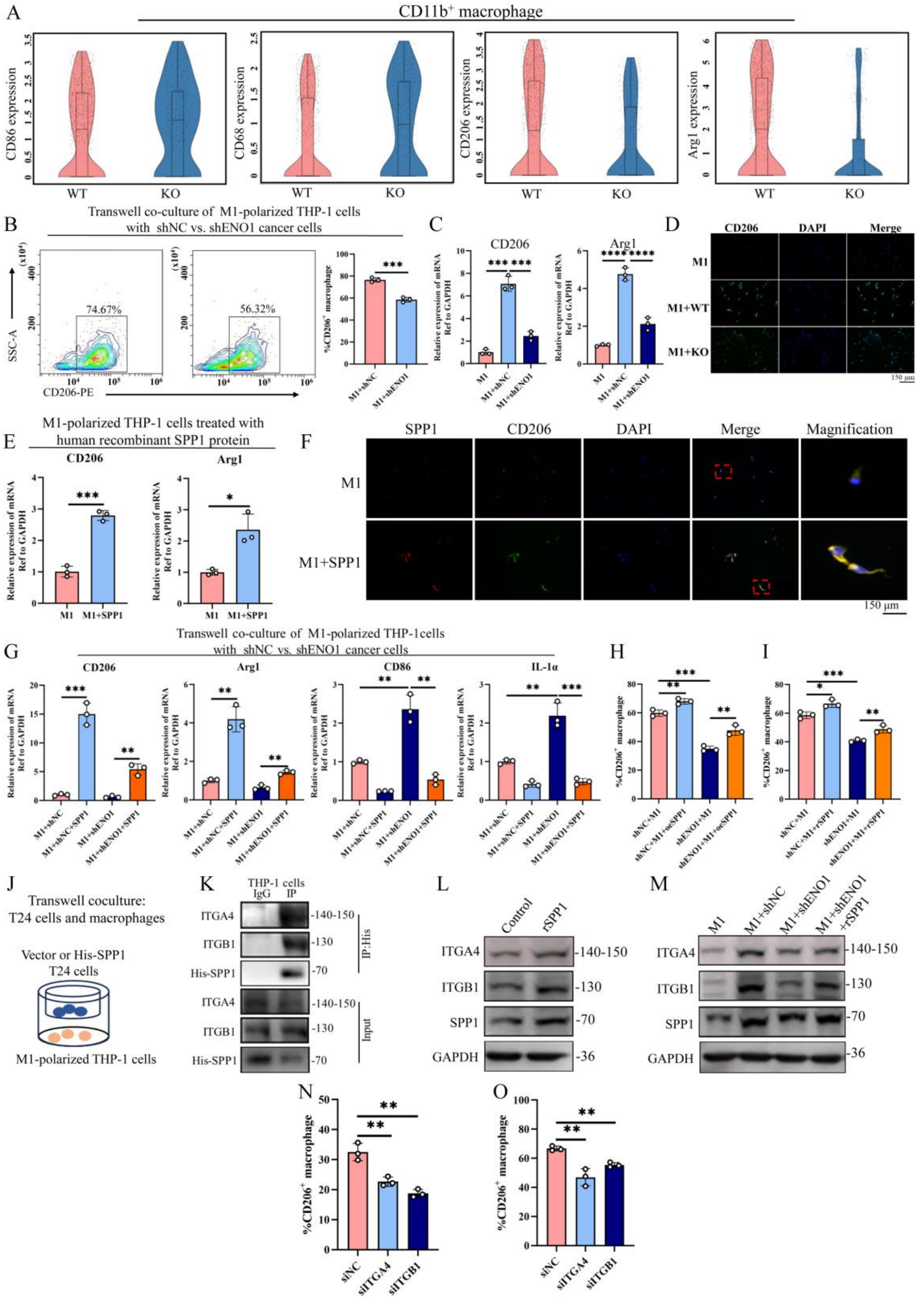

Additional file4. ENO1 promotes the polarization of M2 TAMs via SPP1 in TME. **(A)** The expression levels of M1-like TAM marker genes (CD86, CD68) and M2-like TAM marker genes (CD206, Arg1) in CD11b$^+$ macrophage. **(B)** Flow cytometric analysis of CD206 expression in M1-polarized THP-1 cells cocultured with shNC or

shENO1 cells. n = 3 biologically independent samples per group. Two-side unpaired Student's t-test. Data are presented as mean ± SD. **(C)** The mRNA expression of M2 TAM-like marker gene (CD206, Arg1) in M1-polarized THP-1 cells cocultured with shNC or shENO1 cells. n = 3 biologically independent samples per group. Two-side unpaired Student's t-test. Data are presented as mean ± SD. **(D)** Immunofluorescence analysis of CD206 expression in M1-polarized BMDMs cocultured with WT or ENO1-KO cells. n = 3 biologically independent samples per group. **(E)** The mRNA expression of M2 TAM-like marker gene (CD206, Arg1) in M1-polarized THP-1 cells cocultured with or without human SPP1 recombinant protein. n = 3 biologically independent samples per group. Two-side unpaired Student's t-test. Data are presented as mean ± SD. **(F)** Immunofluorescence analysis for CD206 expression for M1-polarized BMDMs cocultured with or without mouse SPP1 recombinant protein. n = 3 biologically independent samples per group. **(G)** The mRNA expression of M2 TAM-like marker gene (CD206, Arg1) and M1-like marker gene (CD86, IL-1α) in M1-polarized THP-1 cells cocultured with shNC or shENO1 cells, with or without SPP1 overexpression. n = 3 biologically independent samples per group. One-way ANOVA with Dunnett's multiple comparisons test. Data are presented as mean ± SD. **(H, I)** Flow cytometric analysis of CD206 expression in M1-polarized THP-1 cells cocultured with WT or ENO1-KO cells, with or without SPP1 overexpression or recombinant mouse SPP1 protein. n = 3 biologically independent samples per group. One-way ANOVA with Dunnett's multiple comparisons test. Data are presented as mean ± SD. **(J)** A schematic representation of a transwell coculture assay involving ex vivo programmed M1-polarized THP-1 cells combined with Vector or His-SPP1 T24 cells. **(K)** Co-immunoprecipitation assays of M1-polarized THP-1 cells were performed using anti-His antibody. n = 3 biologically independent samples per group. **(L)** Western blotting assay detected ITGA4 and ITGB1 protein expression in M1-polarized THP-1 with or without recombinant mouse SPP1 protein treatment. **(M)** Western blotting assay detected ITGA4 and ITGB1 protein expression in M1-polarized BMDMs cocultured with WT or ENO1-KO cells, with or without recombinant mouse SPP1 protein. n = 3 biologically independent samples per group. **(N)** Flow cytometric analysis of CD206

expression in siITGA4 or siITGB1 M1-polarized THP-1 cells treated with recombinant mouse SPP1 protein. n = 3 biologically independent samples per group. **(O)** Flow cytometric analysis of CD206 expression in siITGA4 or siITGB1 M1-polarized THP-1 cells cocultured with T24 cells. n = 3 biologically independent samples per group. Two-side unpaired Student's t-test. Data are presented as mean ± SD. **p < 0.01, ***p < 0.001, ****p < 0.0001.

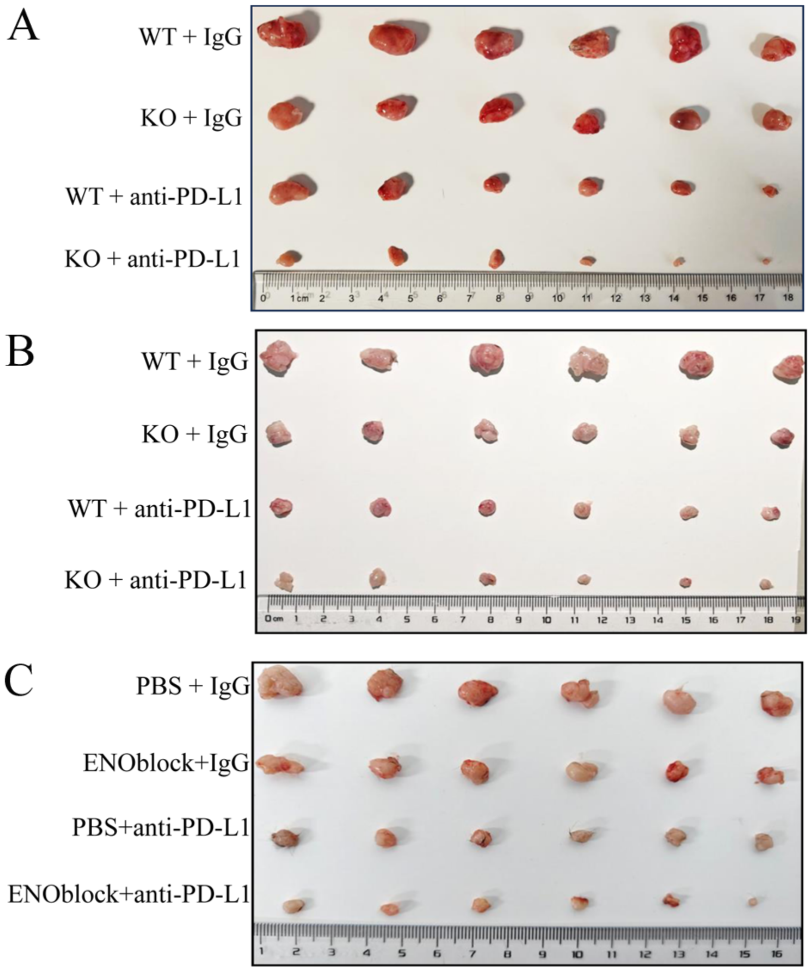

Additional file5. (A) C57BL/6J mice (n=6 per group) were subcutaneously inoculated with WT or ENO1-KO cells. Mice received intraperitoneally treated with 200μg anti-PD-L1 or IgG on days 7, 9, 11, 13, and 15 after tumor inoculation. Tumor sizes were measured. **(B)** In vivo bioluminescence imaging of bladder orthotopic tumor model. Luciferase-labeled WT or ENO1 MB49 cells were subcutaneously inoculated into the bladder of C57BL/6J mice (n=6 per group). Mice were intraperitoneally treated with 200μg anti-PD-L1 or IgG on days 7, 9, 11, and 13 after tumor inoculation. Tumor sizes were measured. **(C)** MB49 cells were subcutaneously inoculated into C57BL/6J mice

and then treatment with ENOblock and anti-PD-L1 (n=6 per group). Tumor sizes were measured.
